# Grafted human ESC-derived astroglia repair spinal cord injury via activation of host anti-inflammatory microglia in the lesion area

**DOI:** 10.7150/thno.70929

**Published:** 2022-05-16

**Authors:** Jian Wang, Peng Jiang, Wenbin Deng, Yuhui Sun, Yaobo Liu

**Affiliations:** 1 Jiangsu Key Laboratory of Neuropsychiatric Diseases and Institute of Neuroscience, Soochow University; Clinical Research Center of Neurological Disease, The Second Affiliated Hospital of Soochow University, Suzhou 215123, China.; 2 Co-innovation Center of Neuroregeneration, Nantong University, Nantong 226001, China.; 3 Department of Orthopedics, Shanghai Tenth People's Hospital, Tongji University School of Medicine, Shanghai 200072, China.; 4 Department of Cell Biology and Neuroscience, Rutgers University, Piscataway, NJ 08854, United States.; 5 School of Pharmaceutical Sciences (Shenzhen), Sun Yat-sen University, Shenzhen, Guangdong 518000, China.

**Keywords:** astroglial transplantation, anti-inflammatory shift of microglia, scar formation, axon regeneration, functional recovery

## Abstract

Grafted astroglia/astrocytes exhibit neuroprotective effects and improve functional recovery after injury to the central nervous system. This study sought to elucidate their ability to repair spinal cord lesions and the underlying mechanisms.

**Methods:** Complete spinal transection, transplantation of astroglia generated from human ESC-derived neural progenitor cells (NPC-Astros) or Olig2-GFP knock-in progenitors (Olig2PC-Astros), and immunostaining were used to determine the survival of astroglia. CUBIC tissue-clearing, immunostaining, electromyography, and functional tests such as the Basso Mouse Scale score and gait analysis were applied to analyze the recovery of the lesion area, axon regeneration, synapse formation, and motor function. Sholl analysis, immunostaining, depletion of anti-inflammatory microglia, and western blotting were employed to explore the cellular and molecular mechanisms underlying spinal cord repair.

**Results:** Grafted NPC- or Olig2PC-Astros survived in the lesion area and assisted wound healing by reducing scar formation and promoting regrowth of descending serotonergic axons and synapse reformation beyond the lesion area. These positive effects resulted in increased Basso Mouse Scale scores and improved hindlimb function as determined by electromyography and gait analysis. Activated microglia in the lesion area were shifted towards an anti-inflammatory phenotype after transplantation of NPC- or Olig2PC-Astros, and depletion of anti-inflammatory microglia reversed the observed improvements in the lesion area and axon regeneration. Transplantation of NPC- or Olig2PC-Astros elevated the expression of interleukin-4 and promoted the phenotypic shift of microglial via interleukin-4 downstream signaling.

**Conclusion:** Our findings indicate that grafted human ESC-derived NPC- or Olig2PC-Astros promote recovery of the injured spinal cord by shifting microglia towards an anti-inflammatory state in the lesion area and activating interleukin-4 signaling.

## Introduction

Astroglia/astrocyte transplantation is a promising strategy for repairing injuries to the central nervous system. Grafting of astrocytes, such as those derived from human induced pluripotent stem-cells 1, astrocytes expressing BDNF or GDNF 2-4, NPC-Astros, and Olig2PC-Astros derived from human embryonic stem cells 5, increases neuronal survival, improves learning and memory, and reduces motor deficits in patients with amyotrophic lateral sclerosis, Parkinson's disease, ischemic stroke, and Alzheimer's disease. Embryonic, neonatal, and adult cortical astrocytes 6-9, induced pluripotent stem cell-derived astrocytes 10, astrocytes derived from embryonic glial-restricted precursors 11, mouse/rat/human astrocytes derived from BMP-treated glial-restricted precursors (GDAs^BMP^), and CNTF-treated glial-restricted precursors (GDAs^CNTF^) 12-14 have all been reported to positively change the inhibitory environment at the lesion site, provide support for axon regrowth, and promote functional recovery after spinal cord injury (SCI). Despite the exciting progress in this area, many issues remain to be addressed, including which cellular and molecular mechanisms underlie the repair of SCI by grafted astroglia/astrocytes.

Scar formation can be either beneficial or detrimental to recovery after a SCI. On the one hand, glial scars form around the lesion area and protect nearby healthy tissue 15. On the other hand, scar formation creates a compact barrier that prevents axon regeneration and functional synapse formation 16-18. Growing evidence shows that grafting astrocytes, such as those from newborn rat cerebral cortex 7, human GDAs^BMP^ 14, and rat GDAs^BMP^ 19, 20 decreases injury size and reduces scarring after SCI. Furthermore, reducing pericytes in the lesion area 21, reducing glial scarring 22, or reducing cyst volume 23 can promote axon regeneration and functional recovery after SCI, indicating that reduction of the lesion area or scar tissue promotes repair. NPC-Astros and Olig2PC-Astros exhibit superior neuroprotective effects and improve behavioural outcome in ischemic stroke 5. However, it is not known whether transplantation of NPC- and Olig2PC-Astros to a SCI can prevent scarring or assist functional recovery.

Microglia, which are the resident immune cells in the central nervous system, undergo rapid proliferation and a shift towards both pro- and anti-inflammatory phenotypes in response to SCI 24. Pro- and anti-inflammatory microglia play different and important roles in the progression of SCI and its repair. Activated microglia form a protective barrier that limits further infiltration and promotes wound compaction to minimize scar size 25, 26. Depletion of microglia disrupts scar formation and is harmful to lesion repair 24, 27. Neonatal microglia or, alternatively, adult microglia that have been treated with peptidase inhibitors facilitate scar-free healing and axon regeneration 28. Anti-inflammatory microglia promote tissue repair and recovery of motor function after SCI 29, 30. Several lines of evidence indicate that crosstalk between astrocytes and microglia is essential for central nervous system homeostasis as well as disease-related neuroinflammation. For instance, neurotoxic reactive A1 astrocytes can be induced by activated microglia 31, astrocytes remove cell debris when microglial phagocytic activity is impaired 32, and microglia can be reprogrammed by astrocyte-derived interleukin-3 (IL-3) to ameliorate the pathology of Alzheimer's disease 33. Therefore, we were interested in whether transplantation of astroglia after SCI could affect microglial activation and polarization—especially in the lesion area.

NPC-Astros or Olig2PC-Astros are specific subtypes of astroglia that are generated from human embryonic stem cells, as Jiang et al reported 5. They were generated from Olig2^-^/GFP^-^ (negative) NPCs and Olig2^+^/GFP^+^(positive) progenitor cells (Olig2PCs) that derived from Olig2-GFP negative and Olig2-GFP knockin hESCs, respectively. NPC-Astros or Olig2PC-Astros exhibited neuroprotective effects in cerebral ischemia model 5, and that human iPSC-derived immature astroglia (hiPSC-Astros) promote myelinogenesis and improve behavioral outcomes in a rat model of periventricular leukomalacia 34. Here we investigate whether these two specific subtypes of astroglia play roles in spinal cord injury and reveal the alternative cellular and molecular mechanisms underlying NPC/Olig2PC-Astros repair injured spinal cord. Our study provides new insights into subsequent cell therapies for spinal cord injury, namely, grafted astroglia promote the host microglia shift to anti-inflammatory microglia to repair injured spinal cord.

## Results

### Astroglia transplantation into injured mouse spinal cord

**Figure 1:**
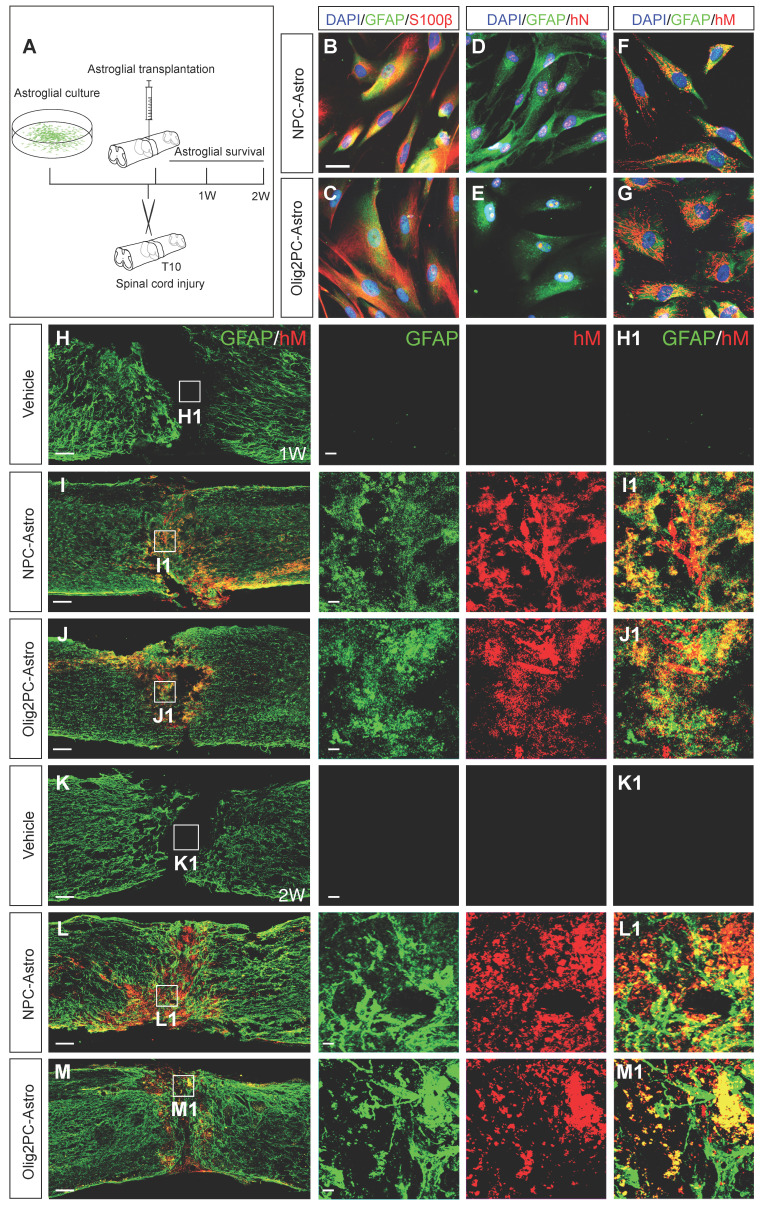
** Culture and transplantation of NPC- and Olig2PC-Astros. (A)** Transplantation strategy and detection. **(B-G)** Co-staining for GFAP (green) and S100β (red) and staining of human nuclei (hN, red) and human mitochondria (hM, red) in cultured NPC- and Olig2PC-Astros. Blue: DAPI-stained nuclei. Scale bars: 20 μm. **(H-M)** Representative images showing that NPC-Astros **(I, L)** and Olig2PC-Astros **(J, M)** were successfully transplanted as characterized by GFAP (green) and hM (red) staining 1 week **(H-J)** and 2 weeks **(K-M)** after transplantation. **H1-M1** are higher magnification images of boxed areas in **H-M**. Scale bars: 100 μm **(H-M)**, 20 μm **(H1-M1)**.

Cultured NPC- and Olig2PC-Astro cells derived from human embryonic stem cells were passaged 3-10 times prior to transplantation. The stability of both cell types was confirmed by immunostaining for the astroglial markers GFAP (**Figure 1B-G**) and S100β (**Figure 1B, C**) and for human nuclei (hN; **Figure 1D, E**) and human mitochondria (hM; **Figure 1F, G**). Nuclei were stained with DAPI (**Figure 1B-G**). These results indicated that the cells retained their expected characteristics after being passaged up to 10 times in culture.

Complete transection was performed at the T10 spinal cord segment of 8- to 10-week-old mice, and NPC- and Olig2PC-Astros were injected into the lesion epicenter 1 day after injury (**Figure 1A**). Co-immunostaining with antibodies against human mitochondria and GFAP revealed that NPC- and Olig2PC-Astros were successfully grafted and survived in the lesion area for at least 2 weeks after transplantation (**Figure 1L, L1, M, M1**). Co-staining of GFAP and hM was detected in the lesion center 1 week (**Figure 1I, I1, J, J1**) and 2 weeks (**Figure 1L, L1, M, M1**) after NPC- and Olig2PC-Astro transplantation, whereas neither marker was detected in the vehicle group (**Figure 1H, H1, K, K1**). At 2 weeks post-transplantation, most grafted astroglia remained in the lesion center (**Figure 1L1, M1**), although some migrated more than 1 mm beyond the lesion border and integrated into rostral and caudal spinal cord regions (**Figure 1L, M**).

### Grafted astroglia reduce the lesion area

**Figure 2:**
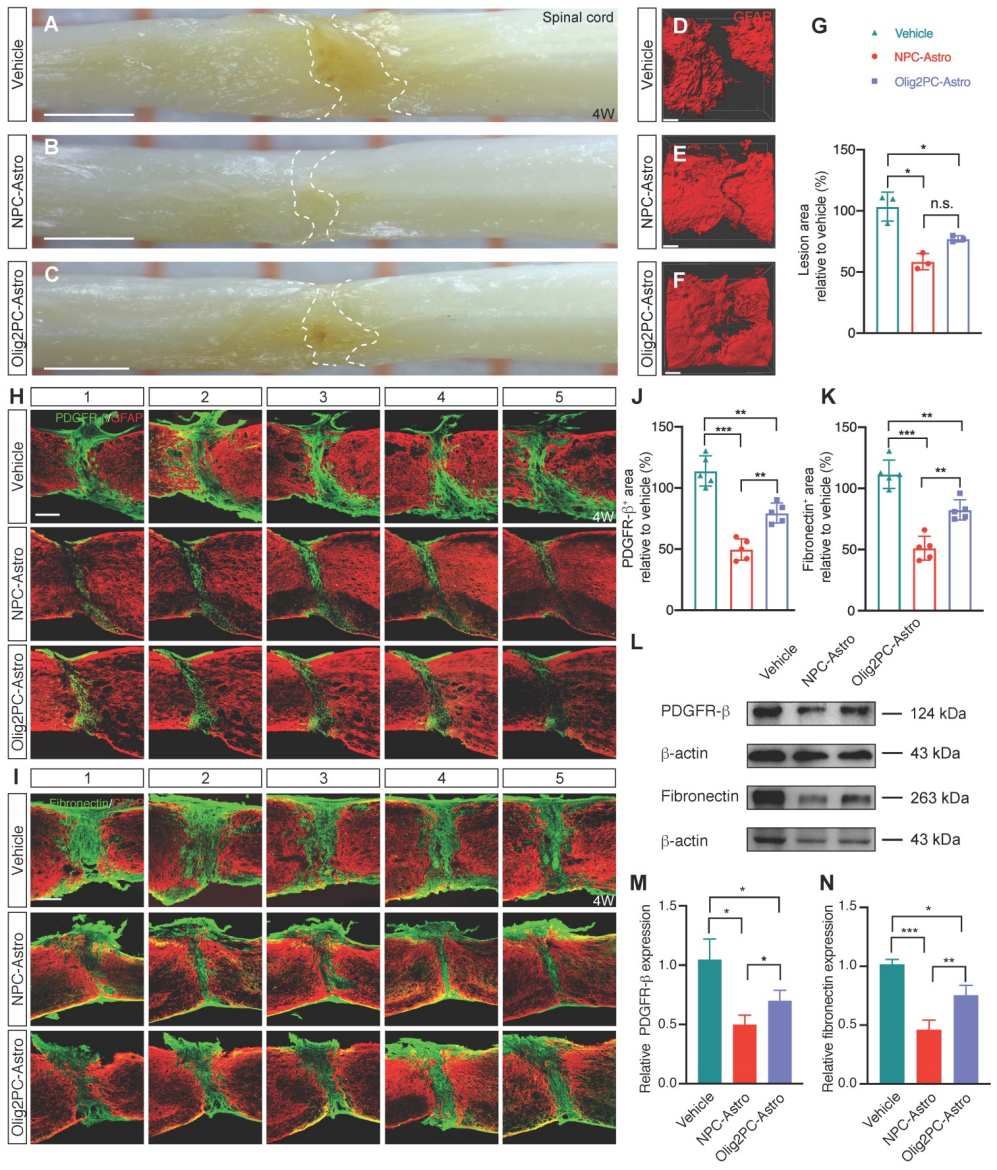
** Transplantation of NPC- and Olig2PC-Astros reduces SCI lesion areas. (A-C)** Lesion area in representative vehicle, NPC-Astro, Olig2PC-Astro samples at 4 weeks post-transplantation. Scale bars: 1000 μm. **(D-F)** Lesion area in representative GFAP-labeled vehicle, NPC-Astro, Olig2PC-Astro spinal cord samples at 4 weeks post-transplantation. Scale bars: 200 μm.** (G)** Quantification of lesion area in vehicle, NPC-Astro, and Olig2PC-Astro groups. Data are mean ± SD; *n* = 3 per group. Brown-Forsythe and Welch ANOVA followed by Dunnett's T3 multiple comparisons test. **(H, I)** Representative five consecutive parasagittal spinal cord sections around the midline at 4 weeks post-transplantation, stained for GFAP (red) and either PDGFR-β **(H)** or fibronectin **(I)** (green). Scale bars: 200 μm. **(J, K)** Quantification of PDGFR-β^+^ **(J)** and fibronectin^+^ **(K)** area, respectively. Data are mean ± SD; *n* = 5 per group. Brown-Forsythe and Welch ANOVA followed by Dunnett's T3 multiple comparisons test. **(L)** Western blotting for PDGFR-β and fibronectin protein expression at 4 weeks post-transplantation.** (M, N)** Quantification of PDGFR-β and fibronectin expression from western blots. Data are mean ± SD; *n* = 5 per group. Brown-Forsythe and Welch ANOVA followed by Dunnett's T3 multiple comparisons test. For all graphs, *p < 0.05, **p < 0.01, ***p < 0.001, n.s., not significant.

To examine the effects of grafted astroglia on scar formation 4 weeks after experimentally induced SCI, lesioned spinal cords were first visualized with a digital surgical microscope (**Figure 2A-C**) and then cleared, labeled with anti-GFAP-Cy3, and visualized with a confocal microscope (**Figure 2D-F**). Compared with the vehicle group, the lesion area in the NPC- and Olig2PC-Astro groups decreased by 45.03% (*p* < 0.05) and 26.37% (*p* < 0.05), respectively (*n* = 3 per group; **Figure 2G**).

Scar formation involves recruitment of various cell types, including pericytes and fibroblasts, that secrete and lay down the extracellular matrix components of scar tissue. To visualize pericytes and fibroblasts at the lesion site, parasagittal sections were co-immunostained for GFAP and PDGFR-β or fibronectin 4 weeks after transplantation (**Figure 2H, I**). The area containing pericytes/fibroblasts was measured in five consecutive sagittal sections centered at the midline. Compared with the vehicle group, the area containing PDGFR-β^+^ cells in the NPC- and Olig2PC-Astro groups was reduced by 64.2% (*p* < 0.001) and 34.4% (*p* < 0.01), respectively (*n* = 5 per group, **Figure 2J**). The average PDGFR-β^+^ area for the NPC-Astro group was 29.8% lower than that of the Olig2PC-Astro group (*p* < 0.01). A similar trend was seen for average fibronectin^+^ area (**Figure 2K**). Compared with the vehicle group, the fibronectin^+^ area of the NPC- and Olig2PC-Astro groups was reduced by 61.8% (*p* < 0.001) and 30.6% (*p* < 0.01), respectively (*n* = 5 per group), and the fibronectin^+^ area of the NPC-Astro group was 31.2% lower than that of the Olig2PC-Astro group (*p* < 0.01). Western blotting was performed to verify PDGFR-β and fibronectin expression in the lesion area 4 weeks after transplantation (**Figure 2L**). Compared with the vehicle group, the NPC-Astro and Olig2PC-Astro groups had significantly reduced basal levels of both proteins (*n* = 5 per group, *p* < 0.05 in **Figure 2M,** *p* < 0.001 and 0.05 in **Figure 2N**). These results demonstrated that grafted astroglia can reduce the lesion area after SCI. Microglia has been reported to play a key role on scar healing and lesion area reduction after SCI 24, 28. NPC-Astros and Olig2PC-Astros as xenografts derived from human embryonic stem cells may recruit immune cells such as microglia to the lesion area or activate those immune cells, so as to promote the lesion area reduction after SCI. Further, we explored the response of host microglia after astroglia transplantation following SCI.

### Grafted astroglia promote serotonergic axon regrowth and synapse reformation

**Figure 3:**
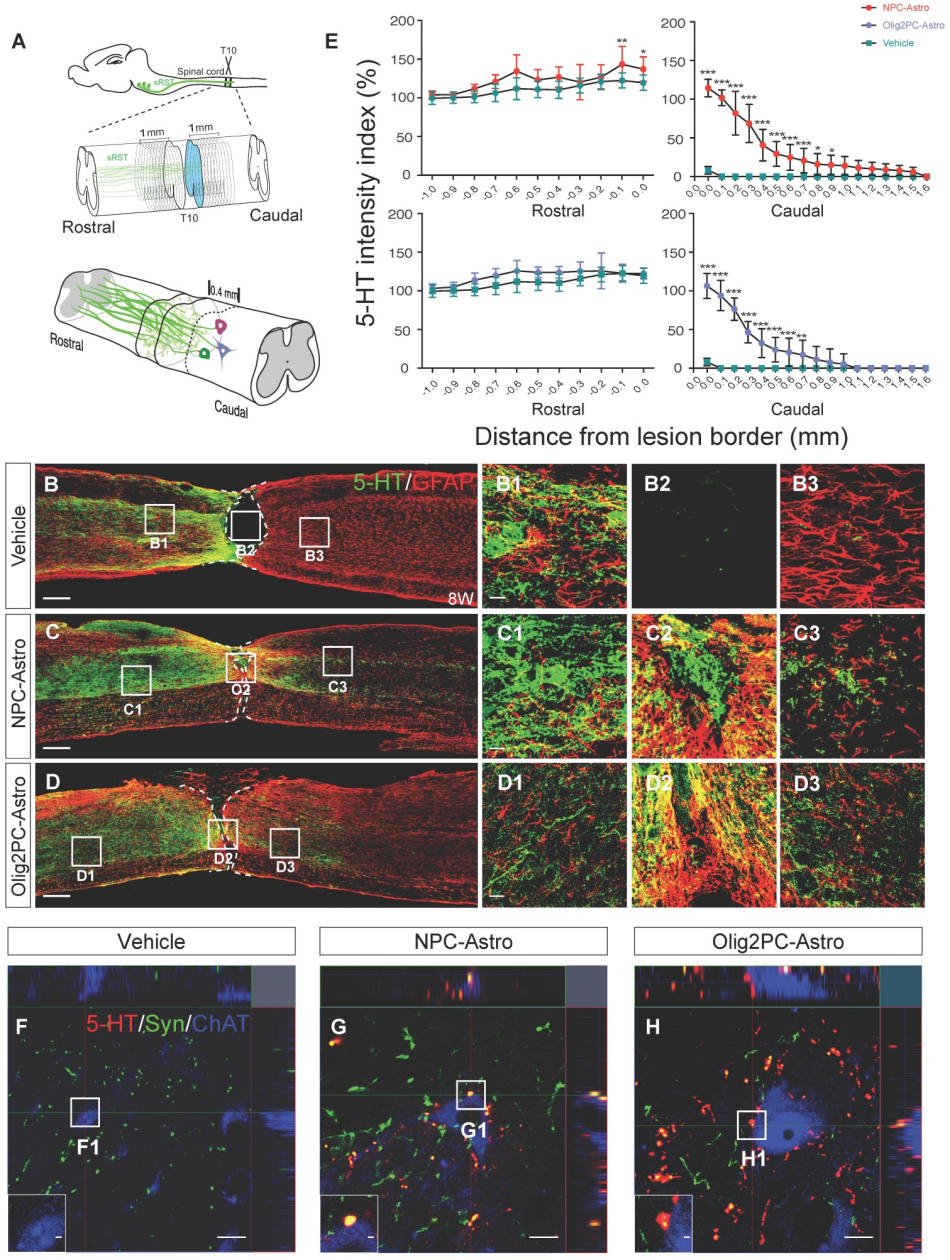
** Grafted NPC- and Olig2PC-Astros promote serotonergic axon regeneration and synapse formation after SCI. (A)** Schematic of quantification strategy for serotonergic axon regeneration (top) and synapse formation (bottom) in the caudal spinal cord. sRST, serotonergic raphespinal tract. **(B-D)** Representative images showing co-staining for 5-HT (green) and GFAP (red) in spinal cord sagittal sections from vehicle, NPC-Astro, and Olig2PC-Astro groups at 8 weeks post-transplantation. **B1-B3**, **C1-C3**, and **D1-D3** are higher magnification images of boxed areas in **B-D**. Dashed lines indicate lesion borders. Scale bars: 200 μm **(B-D)**, 20 μm **(D1-D3)**. **(E)** Quantification of serotonergic axons rostral and caudal to lesion sites in vehicle (*n* = 9), NPC-Astro (*n* = 12), and Olig2PC-Astro (*n* = 10) groups. Two-way ANOVA followed by Tukey's multiple comparisons test. Data are mean ± SD; *p < 0.05, **p < 0.01, ***p < 0.001. **(F-H)** Z-stacked images of triple immunostaining in vehicle, NPC-Astro, and Olig2PC-Astro groups. **F1-H1** are higher magnification images of boxed areas in **F-H**. In **G1** and **H1**, regenerated serotonergic axons (5-HT, red) co-localized with synaptophysin (green), which appears to be directly associated with host motor neurons (ChAT, blue). Scale bars: 10 μm **(F-H)**, 1 μm** (F1-H1)**.

Because axons from the descending serotonergic raphespinal tract are critical for hindlimb locomotor recovery after SCI 35, 36, we next examined the effects of astroglia on regeneration of serotonergic axons 8 weeks after transplantation (**Figure 3A**). Axon growth was examined by immunofluorescence labeling for 5-HT. At 8 weeks, most serotonergic axons in the vehicle group had retracted from the lesion borders, with few axons extending into or across the lesion site (**Figure 3B, B1-B3**). Conversely, large numbers of serotonergic axons in the astroglial transplantation groups grew across the lesion area and into the caudal spinal cord (**Figure 3C, D, C1-C3, D1-D3**). Specifically, serotonergic axons in the NPC-Astro and Olig2PC-Astro groups extended ~1.5 and ~0.9 mm caudal to the lesion site, respectively. Furthermore, the regenerated axons extended in association with scar-forming astrocytes, suggesting a supportive role for astroglia (**Figure 3C, D, C1-C2, D1-D2**). To quantify the observed regrowth, we measured across two 1-mm regions, one extending rostrally from the rostral lesion border, the other extending caudally from the caudal lesion border (**Figure 3A**). Compared with vehicle (*n* = 9, **Figure 3E**), 5-HT intensity was significantly greater for both the NPC-Astro group **(**-0.1 to +0.0 mm, *p* < 0.01 to 0.05; +0.0 mm to +0.7 mm, *p* < 0.001; +0.8 mm to +0.9 mm, *p* < 0.05; *n* = 12) and Olig2PC-Astro group **(**0.0 mm to +0.6 mm, *p* < 0.001; +0.7 mm, *p* < 0.01; *n* = 10). These observations suggested that NPC- and Olig2PC-Astros promoted serotonergic axon regrowth across the lesion site.

We found that, rostral to the lesion site, 5-HT levels are highest closest to the lesion site, which might indicate serotonergic neurons are searching for suitable synaptic targets. To determine whether regenerated serotonergic axons integrated into the neural circuitry and identify the types of neurons innervated, we performed triple immunostaining for 5-HT, synaptophysin (a presynaptic marker), and ChAT (a motor neuron marker) within 0.4 mm caudal of the lesion site (**Figure 3A**). In the vehicle group, few 5-HT^+^ axons and synaptophysin^+^ structures were detected (**Figure 3F, F1**). In the NPC- and Olig2PC-Astro groups, 5-HT^+^ axons were associated with synaptophysin and formed bouton-like terminals around the cell bodies of ChAT positive motor neurons (**Figure 3G-H, G1-H1**), suggesting that synapses between serotonergic axons and motor neurons reformed after NPC- and Olig2PC-Astro transplantation.

### Grafted astroglia improve locomotive function and electrophysiological transmission

**Figure 4:**
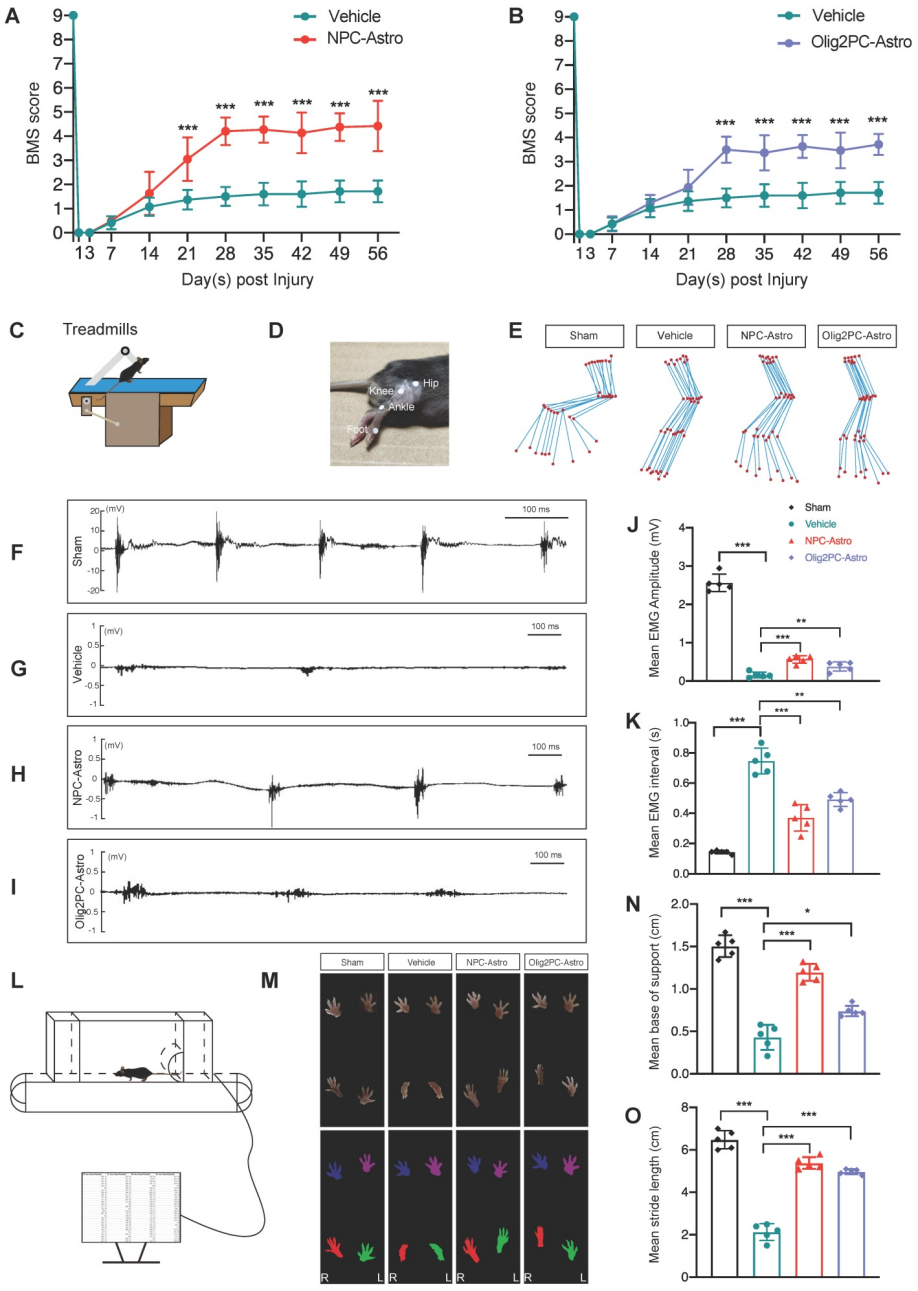
** Grafted astroglia improve locomotive function and electrophysiological transmission after SCI. (A, B)** BMS scores from open-field tests for hindlimb locomotion performed weekly after transection (*n* = 10 per group). Two-way ANOVA followed by Bonferroni's multiple comparisons test. Data are mean ± SD; ***p < 0.001. **(C)** Schematic of EMG analysis of the right hindlimb TA muscle to test electrophysiological transmission 4 weeks after astroglial transplantation. **(D)** The hip, knee, ankle, and foot joints of the right hindlimb were marked by small discs. **(E)** Right hindlimb kinematics and hindlimb end-point trajectory. **(F-I)** EMGs of sham, vehicle, NPC-Astro, and Olig2PC-Astro mice. **(J, K)** Mean EMG amplitude **(J)** and mean EMG interval** (K)** in sham, vehicle, NPC-Astro, and Olig2PC-Astro mice (*n* = 5 per group). **(L)** Schematic of gait analysis performed at 4 weeks post-transplantation. **(M)** Footprints of sham, vehicle, NPC-Astro, and Olig2PC-Astro mice. **(N, O)** Mean base of support **(N)** and stride length **(O)** in sham, vehicle, NPC-Astro, and Olig2PC-Astro mice (*n* = 5 per group). For **(J, K, N, O)**: Brown-Forsythe and Welch ANOVA followed by Dunnett's T3 multiple comparisons test. Data are mean ± SD; *p < 0.05, **p < 0.01, ***p < 0.001.

To determine whether the observed regeneration was associated with changes in neuronal function, we first examined the locomotive status of mice using the BMS. Compared with vehicle, BMS scores improved significantly for mice that received NPC-Astros or Olig2PC-Astros (**Figure 4A, B**; *n* = 10 per group). Increased locomotive activity was significant from 21 days (*p* < 0.001 for NPC-Astro) or 28 days (*p* < 0.001 for Olig2PC-Astro) after injury until the end of testing at 56 days (*p* < 0.001 for NPC-Astro, *p* < 0.001 for Oligo2PC-Astro).

In addition, EMG activity of the right hindlimb TA muscle was evaluated 4 weeks after transplantation by observing sham, vehicle, NPC-Astro, and Olig2PC-Astro mice on a treadmill (**Figure 4C**). The hip, knee, ankle, and foot joints of the right hindlimb were marked by small discs (**Figure 4D**), locomotion was captured with a camera, and motion trajectories were reconstructed (**Figure 4E**). Mice in the sham group (laminectomy only) were able to move forward voluntarily on the treadmill, whereas those in the vehicle group were essentially motionless. Mice in the NPC- and Olig2PC-Astro groups moved voluntarily on the treadmill and had hindlimb trajectories similar to those of the sham group (**Figure 4E**). Next, an electrode for real-time surface EMG was fettered to the surface of the TA muscle to record muscle activity during voluntary movement on the treadmill. Mice in the sham group demonstrated rhythmic muscle movement (**Figure 4F**), whereas those in the vehicle group demonstrated little muscle activity (**Figure 4G**). Mice in the NPC- and Olig2PC-Astro groups (**Figure 4H, I**) demonstrated some rhythmic muscle movement. The mean EMG amplitude was significantly higher for both transplantation groups than for the vehicle group (*n* = 5 per group, *p* < 0.001 and 0.01, **Figure 4J**), and the EMG interval was significantly shorter (*p* < 0.001 and 0.01, **Figure 4K**). These results indicated that transplantation of NPC- and Olig2PC-Astros led to partial recovery of rhythmic movement in the hindlimb of transected mice.

To further evaluate the recovery of motor function, gait analysis was performed on a treadmill 4 weeks after transplantation (**Figure 4L**). Mouse footprints were captured, and the gait was reconstructed. Hindlimb footprints were abducent for the sham group but were folded and valgus for the vehicle group (**Figure 4M**). Hindlimb footprints for the NPC- and Olig2PC-Astro groups partially recovered so as to mimic those of the abducent state of the sham group (**Figure 4M**). Forelimb footprints of all groups were abducent, as expected. To quantify the footprint data acquired during the gait analysis, we examined base of support and stride length (**Figure 4N, O**). Both parameters decreased significantly for the vehicle group compared with the sham group (*n* = 5 per group, *p* < 0.001) but increased significantly for both transplantation groups relative to the vehicle group (*n* = 5 per group, *p* < 0.05 and 0.001). These results demonstrated that grafted astroglia promoted the recovery of gait after SCI and combine with the BMS and EMG results to support recovery of motor function on multiple levels.

### Grafted astroglia promote anti-inflammatory polarization of microglia

Microglia perform a variety of functions depending on whether they express a pro-inflammatory (M1) or anti-inflammatory (M2) phenotype 37, a phenomenon known as microglial polarization. Because anti-inflammatory microglia are involved in repair of tissue damage 29, 30, we investigated whether astroglial transplantation could affect the proliferation and polarization of microglia following SCI.

Previous study has revealed that the density of microglia at 0.8 to 1.2 mm (rostral or caudal to the lesion center) did not show significant difference from 4 to 35 days after spinal cord injury 24. In our study, microglia at rostral or caudal 1 mm to the lesion center in the spinal cord showed no difference in the total number and morphology in the vehicle, NPC-Astro and Olig2PC-Astro groups 4 weeks after astroglia transplantation (**Figure S1A, B, C**). To further describe the microglial response to spinal cord injury and astroglia transplantation, we analyzed the number of Iba1+ microglia in a 2.5-mm-long region including the lesion at different time points after astroglia transplantation (**Figure S1D, E, F**). Our results indicated that the density of microglia at rostral or caudal 1 mm to the lesion center showed no difference from 3 to 56 days after astroglia transplantation in all groups (**Figure S1D, E, F**). Therefore, we defined the 1 mm as a critical line to quantify total microglia in a 2-mm-long region including the lesion area.

To further examine the changes in the microglia number after astroglial transplantation, we performed immunolabeling for the microglial marker ionized calcium-binding adaptor molecule 1 (Iba1) (**Figure S2A-C, A1-C1**). We analyzed the total number of Iba1^+^ microglia in a 2-mm-long region including the lesion (total microglia) as well as the number of Iba1^+^ microglia within the GFAP-defined lesion center (microglia in the lesion) on days 3, 7, 14, 28, and 56 after transplantation. All data were normalized as a percentage relative to microglia in the vehicle group at 56 days. For total microglia, there was no significant change for the Olig2PC-Astro group at different time points (*p* > 0.05), and there was no significant change for the NPC-Astro group at different time points (*p* > 0.05) (*n* = 5 for all groups, **Figure S2D**). Similarly, for microglia in the lesion, there was no significant change for the Olig2PC-Astro group at different time points (*p* > 0.05) and no significant change for the NPC-Astro group at different time points (*p* > 0.05) (*n* = 5 for all groups, **Figure S2E**). We next employed western blotting to examine the expression of Iba1 in the lesion area from 3 to 56 days after transplantation (**Figure S2F**). Compared with the vehicle group, the expression of Iba1 was not significantly changed in the NPC-Astro and Olig2PC-Astro groups at 3 to 56 days (n = 3 per group, **Figure S2G**). These results indicated that astroglial transplantation did not promote the increase of host microglia, compared to the vehicle group.

To further examine microglial proliferation, we performed the co-staining of Ki67 and Iba1 to examine whether astroglia transplantation affected the microglial proliferation (**Figure S3A-D**). 3 days after astroglia transplantation, no significant changes were observed in terms of microglial proliferation compared to the vehicle group (**Figure S3G, H**). 7 days after astroglia transplantation, the number of Iba1&Ki67 double positive microglia (**Figure S3I**) and the percentage of Iba1+ microglia undergoing proliferation after SCI was not increased in the astroglia group (**Figure S3J**), compared with the vehicle group. 14, 28, 56 days after astroglia transplantation, only few (1-7%) microglia were still expressing Ki67, there were also no significant changes were observed in terms of microglial proliferation compared to the vehicle group (**Figure S3 Figure S3E, F, K, L, M, N**). Together, these results showed that astroglia transplantation did not promote microglial proliferation, compared to the vehicle group.

To examine microglial polarization after transplantation, we performed double-labeling for Iba1 and either the pro-inflammatory microglial marker iNOS or the anti-inflammatory microglial marker Arg1. 4 weeks after transplantation, there were more iNOS^+^ (pro-inflammatory) microglia in the vehicle group than in the NPC- and Olig2PC-Astro groups (**Figure S4A-C, A1-C1**), but there were more Arg1^+^ (anti-inflammatory) microglia in the NPC- and Olig2PC-Astro groups than in the vehicle group (**Figure S4F-H, F1-H1**). To quantify microglial polarization at different time points, we again examined total microglia (in a 2-mm-long region including the lesion area) and microglia at the lesion (based on GFAP) on days 3, 7, 14, 28, and 56 days after transplantation. All data were normalized as a percentage relative to iNOS^+^ or Arg1^+^ microglia in the vehicle group at 56 days. There was a progressive decrease in total iNOS^+^ microglia beginning at 14 days for the NPC-Astro group (14 days, *p* < 0.05; 28 to 56 days, *p* < 0.001) and 28 days for the Olig2PC-Astro group (28 to 56 days, *p* < 0.01) (*n* = 5 per group, **Figure S4D**). In addition, there was a complementary increase in total Arg1^+^ microglia beginning at 3 days for the NPC-Astro group (3 days and 56 days, *p* < 0.01; 7 to 28 days, *p* < 0.001) and 7 days for the Olig2PC-Astro group (7 days, *p* < 0.05; 14 and 56 days, *p* < 0.001; 28 days, *p* < 0.01) (*n* = 5 per group; **Figure S4I**). A similar trend was observed for microglia at the lesion, with a progressive decrease in iNOS^+^ microglia beginning at 7 days for the NPC-Astro group (7 to 56 days, *p* < 0.001) and 28 days for the Olig2PC-Astro group (28 to 56 days, *p* < 0.01) (*n* = 5 per group; **Figure S4E**), and an increase in Arg1^+^ microglia beginning at 3 days (3 days, *p* < 0.05; 7 to 56 days, *p* < 0.001) for the NPC-Astro group and 7 days for the Olig2PC-Astro group (7 to 14 days, *p* < 0.001; 28 to 56 days, *p* < 0.01) (*n* = 5 per group; **Figure S4J**). These results indicated that astroglia transplantation promoted the phenotypic shift of host microglia.

**Figure 5:**
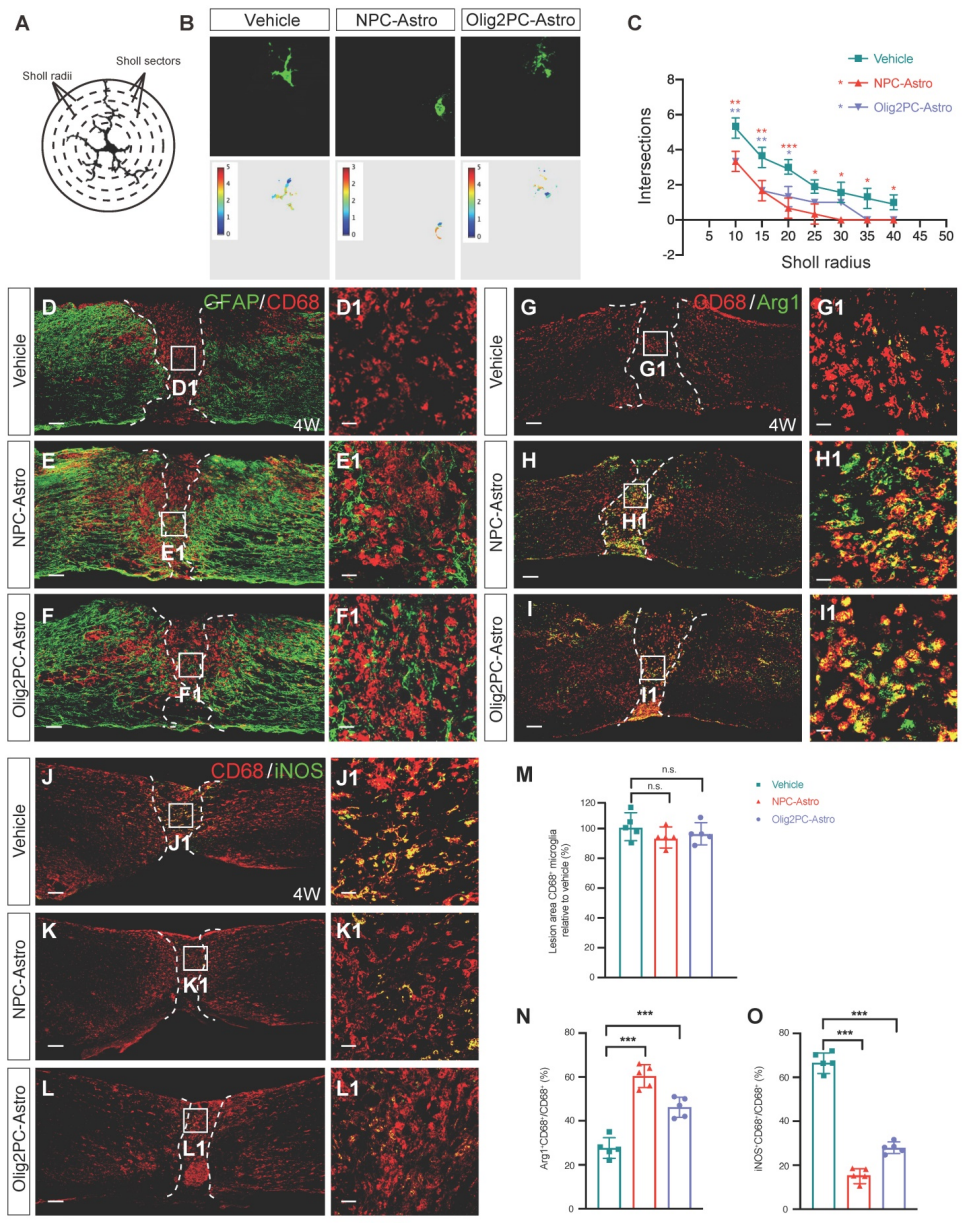
** Grafted astroglia promote the phenotypic shift of microglial. (A)** Diagram of branch intersections in the circular grid.** (B)** Confocal images and pseudocolor representations of microglia in the lesion area. **(C)** Quantification of intersections by Sholl analysis (*n* = 30 per group). Two-way ANOVA followed by Bonferroni's multiple comparisons test. Data are mean ± SD; *p < 0.05, **p < 0.01, ***p < 0.001 versus vehicle. **(D-F)** Representative images showing GFAP (green) and CD68 (red) staining in spinal cord sagittal sections at 4 weeks post-transplantation. **D1-F1** are higher magnification images of boxed areas in **D-F**. Scale bars: 200 μm **(D-F)**, 20 μm **(D1-F1)**. **(M)** Quantification of lesion area CD68^+^ microglia relative to vehicle (*n* = 5 per group). **(G-I)** Representative images showing CD68 (red) and Arg1 (green) staining in spinal cord sagittal sections at 4 weeks post-transplantation. **G1, H1, I1** are higher magnification images of boxed areas in **G-I**. Scale bars: 200 μm **(G-I)**, 20 μm **(G1-I1)**. **(J-L)** Representative images showing CD68 (red) and iNOS (green) staining in spinal cord sagittal sections at 4 weeks post-transplantation. **J1, K1, L1** are higher magnification images of boxed areas in **J-L**. Scale bars: 200 μm **(J-L)**, 20 μm **(J1-L1)**.** (N, O)** Quantification of Arg1^+^CD68^+^ microglia or iNOS^+^CD68^+^ relative to vehicle (*n* = 5 per group). **M, N, O**: Brown-Forsythe and Welch ANOVA followed by Dunnett's T3 multiple comparisons test. Data are mean ± SD; ***p < 0.001, n.s., not significant.

To determine whether grafted NPC- and Olig2PC-Astros could promote activation of host microglia, Sholl analysis was used to evaluate the branching complexity of microglia in the lesion area 4 weeks after transplantation (**Figure 5A**). Images of microglia in the lesion area were captured and converted to pseudocolor images using Image J (**Figure 5B**). In this analysis, microglial branches intersect with a circular grid; fewer intersections are indicative of shorter microglial processes associated with higher branching complexity, which is a characteristic of microglial activation 38. Four weeks after transplantation, the intersections decreased from 10 μm in the vehicle group to 40 μm in the NPC-Astro group and 20 μm in Olig2PC-Astro group (**Figure 5C**), indicating activation of microglia in the lesion center in both astroglial groups.

To further analyze the activation state of microglia after astroglial transplantation, we performed immunolabeling for CD68, a marker of activated microglia. CD68^+^ microglia were detected in the lesion center of the vehicle, NPC-Astro, and Olig2PC-Astro groups 4 weeks after transplantation (**Figure 5D-F, D1-F1**), but the relative number of CD68^+^ microglia was not greater for the NPC-Astro and Olig2PC-Astro groups than for the vehicle group (*n* = 5 per group, *p* > 0.05, **Figure 5M**). This indicated that activated microglia were not proliferated in the lesion center 4 weeks after astroglial transplantation.

Finally, we used CD68 and Arg1 to label both activated and anti-inflammatory microglia (**Figure 5G-I, G1-I1**), CD68 and iNOS to label both activated and pro-inflammatory microglia (**Figure 5J-L, J1-L1**), respectively, and quantified the proportion of CD68^+^ microglia what were also Arg1^+^ or iNOS^+^ 39. In the vehicle group, CD68^+^Arg1^+^ microglia accounted for 27.6 ± 4.72% of total CD68^+^ microglia in the lesion center. In contrast, CD68^+^Arg1^+^ microglia accounted for 60.4 ± 5.18% and 46.2 ± 4.55% of total CD68^+^ microglia in the NPC-Astro group and Olig2PC-Astro group, respectively (*n* = 5 per group, *p* < 0.001 compared with vehicle, **Figure 5N**), indicating that the proportion of anti-inflammatory microglia among activated microglia had increased. In the vehicle group, iNOS^+^CD68^+^ microglia accounted for 66.2 ± 4.21% of total CD68^+^ microglia in the lesion center. In contrast, CD68^+^iNOS^+^ microglia accounted for 17.6 ± 3.71% and 28.5 ± 3.19% of total CD68^+^ microglia in the NPC-Astro group and Olig2PC-Astro group, respectively (*n* = 5 per group, *p* < 0.001 compared with vehicle, **Figure 5O**), indicating that the proportion of pro-inflammatory microglia among activated microglia had decreased. Together, these results suggested that grafted NPC- and Olig2PC-Astros promoted activation of microglia and increased the proportion of activated anti-inflammatory microglia in the lesion center after SCI without changing the total number of activated microglia.

### Depletion of anti-inflammatory microglia inhibits the reduction of the lesion area and regeneration of serotonergic axons

**Figure 6:**
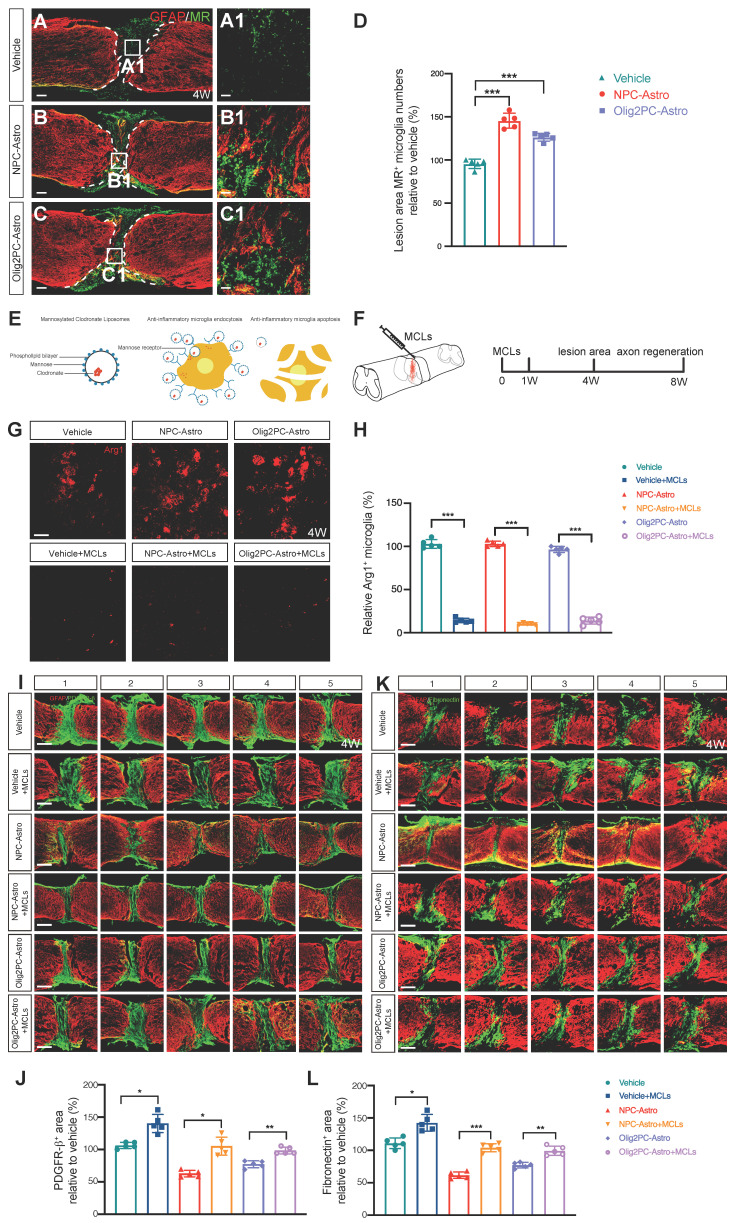
** Depletion of anti-inflammatory microglia inhibits reduction of lesion area. (A-C)** Representative images showing GFAP (red) and MR (green) staining in spinal cord sagittal sections at 4 weeks post-transplantation. **A1-C1** are higher magnification images of boxed areas in **A-C**. Scale bars: 200 μm **(A-C)**, 20 μm **(A1-C1)**. **(D)** Quantification of MR^+^ cells at 4 weeks post-transplantation (*n* = 5 per group). Brown-Forsythe and Welch ANOVA followed by Dunnett's T3 multiple comparisons test. Data are mean ± SD; ***p < 0.001. **(E)** Schematic of depletion of anti-inflammatory microglia using mannosylated clodronate liposomes. **(F)** Experimental design: evaluation of lesion area at 4 weeks and axon regeneration at 8 weeks after MCL injection. **(G)** Representative images showing Arg1(red) staining in sagittal sections of spinal cord lesion area at 4 weeks post-transplantation. Scale bar: 20 μm. **(H)** Quantification of efficiency of anti-inflammatory microglia depletion (*n* = 5 per group). Unpaired t test with Welch's correction. Data are mean ± SD; ***p < 0.001. **(I, K)** Representative images showing five consecutive parasagittal sections centered at the spinal cord midline at 4 weeks post-transplantation. Sections are stained for GFAP (red) to demarcate glial scar borders and lesion areas plus either PDGFR-β to label pericyte (**I**, green) or fibronectin to label fibroblast (**K**, green). Scale bars: 200 μm.** (J, L)** Quantification of PDGFR-β^+^ **(J)** and fibronectin^+^ **(L)** area (*n* = 5 per group). Brown-Forsythe and Welch ANOVA followed by Dunnett's T3 multiple comparisons test. Data are mean ± SD; *p < 0.05, **p < 0.01, ***p < 0.001.

The mannose receptor (MR) is upregulated following anti-inflammatory polarization of microglia 40. Thus, to further assess activation of anti-inflammatory microglia after astroglial transplantation, we used anti-MR immunolabeling to stain anti-inflammatory microglia in the lesion center (**Figure 6A-C, A1-C1**). Four weeks after transplantation, there were significantly more MR^+^ microglia at the lesion center in the NPC-Astro and Olig2PC-Astro groups than in the vehicle group (*n* = 5 per group, *p* < 0.001, respectively, **Figure 6D**), indicating that grafted NPC- and Olig2PC-Astros promoted an increase of MR^+^ microglia in the lesion area.

To evaluate the effects of anti-inflammatory microglia on SCI repair after astroglial transplantation, mannosylated clodronate liposomes (MCLs) were used to deplete anti-inflammatory microglia. As illustrated in **Figure 6E**, MCLs bind to the MR of anti-inflammatory microglia and become absorbed via microglial endocytosis 40, after which the clodronate is released at a concentration that kills the microglia. MCLs were injected into the lesion area with astroglia 1 day after SCI and then injected intraperitoneally once a week for 4 weeks (**Figure 6F**). To determine the efficiency of depletion, we used anti-Arg1 to stain anti-inflammatory microglia 4 weeks after MCLs injection (**Figure 6G**). Quantification of Arg1^+^ microglia revealed depletion by 88.69% in the vehicle + MCLs group, 92.85% in the NPC-Astro + MCLs group, and 82.4% in the Olig2PC-Astro + MCLs group (*n* = 5 per group, *p* < 0.001, **Figure 6H**).

Double-immunostaining for GFAP and either PDGFR-β or fibronectin was performed 4 weeks after transplantation and initial MCLs injection on serial parasagittal sections of injured spinal cord to visualize the glial scar borders and the PDGFR-β^+^ or fibronectin^+^ area, respectively (**Figure 6I, K**). Five consecutive sagittal sections centered at the midline were selected for measurement of PDGFR-β^+^ or fibronectin^+^ areas. Compared with the corresponding groups without MCLs, the average PDGFR-β^+^ area increased by 33.8% in the vehicle + MCLs group, 42.45% in the NPC-Astro + MCLs group, and 21.16% in the Olig2PC-Astro + MCLs group (*n* = 5 per group, *p* < 0.05 and 0.01; **Figure 6J**). Similarly, treatment with MCLs increased the average fibronectin^+^ area by 31.6% in the vehicle + MCLs group, 42.4% in the NPC-Astro + MCLs group, and 22.2% in the Olig2PC-Astro + MCLs group (*n* = 5 per group, *p* < 0.05, 0.001 and 0.01; **Figure 6L**). These results indicated that anti-inflammatory microglia were an essential component of the lesion repair observed after transplantation of NPC- and Olig2PC-Astros.

**Figure 7:**
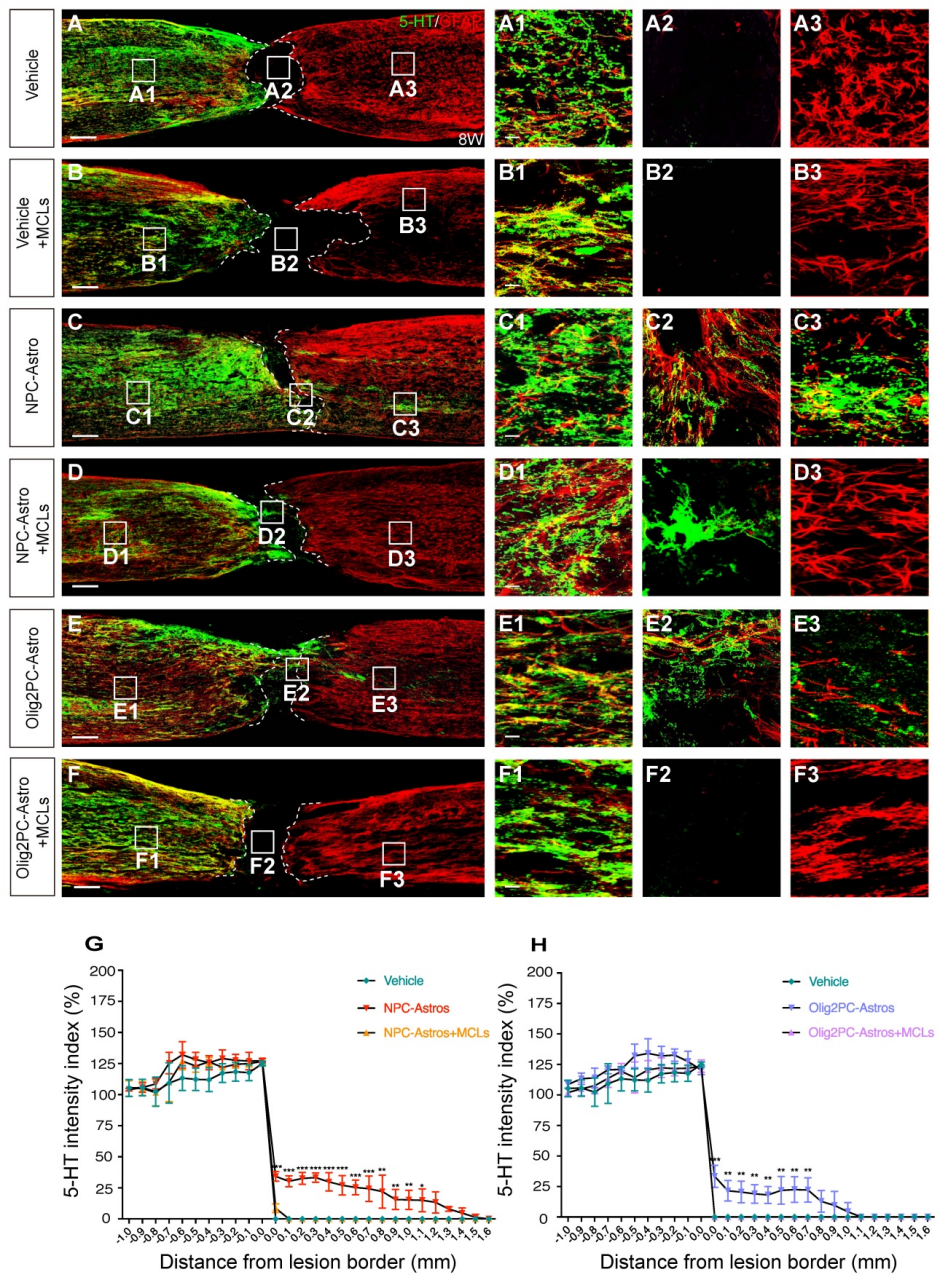
** Depletion of anti-inflammatory microglia inhibits serotonergic axon regrowth. (A-F)** Representative images showing 5-HT (green) and GFAP (red) staining of sagittal spinal cord sections 8 weeks after SCI. **A1-F3** are higher magnification images of boxed areas in **A-F**. Scale bars: 200 μm **(A-F)**, 20 μm **(A1-F3)**. **(G, H)** Quantification of serotonergic axons rostral and caudal to the lesion (*n* = 3 per group). Two-way ANOVA followed by Tukey's multiple comparisons test. Data are mean ± SD; *p < 0.05, **p < 0.01, ***p < 0.001.

Serotonergic axon regrowth was evaluated 8 weeks after astroglial transplantation and initial MCLs injection by double-labeling for GFAP and 5-HT. For the vehicle group, most of the serotonergic axons caudal to the lesion were retracted, with few axons extending into the lesion site (**Figure 7A, A1-A3**). For the NPC- and Olig2PC-Astro groups, more serotonergic axons grew into the lesion area and extended to the caudal spinal cord (**Figure 7C, E, C1-C3, E1-E3**). After MCLs treatment, serotonergic axons were undetectable in the lesion area and caudal spinal cord of the vehicle and Olig2PC-Astro groups (**Figure 7B, F, B1-B3, F1-F3**), and only a few serotonergic axons grew into the lesion of the NPC-Astro group (**Figure 7D, D1-D3**). Notably, there was a larger zone between the rostral and caudal spinal cord in all three MCLs-treated groups as indicated by GFAP. Moreover, host astrocytes were undetectable in the lesion area of the astroglia + MCLs groups, suggesting an inhibitory effect for lesion repair (**Figure 7B, D, F, B2, D2, F2**).

Quantification of serotonergic intensity from -1.0 to +1.6 mm from the rostral lesion border (0 mm) or caudal lesion border (0 mm) revealed a significant decrease in serotonergic axons caudal to the lesion for the NPC-Astro + MCLs group (+0.0 mm to +0.7 mm, *p* < 0.001; +0.8 mm to +1.0 mm, *p* < 0.01; +1.0 mm, *p* < 0.05) and Olig2PC-Astro + MCL group (+0.0 mm, *p* < 0.001; +0.1 mm to +0.7 mm, *p* < 0.01) compared with the NPC- or Olig2PC-Astro groups (*n* **=** 3 per group; **Figure 7G, H**). Taken together, these results indicated that grafted NPC- and Olig2PC-Astros induced the anti-inflammatory polarization of microglia, which reduced the lesion area and established a suitable environment for axon regeneration.

### Grafted astroglia activate anti-inflammatory microglia via IL-4 signaling

**Figure 8:**
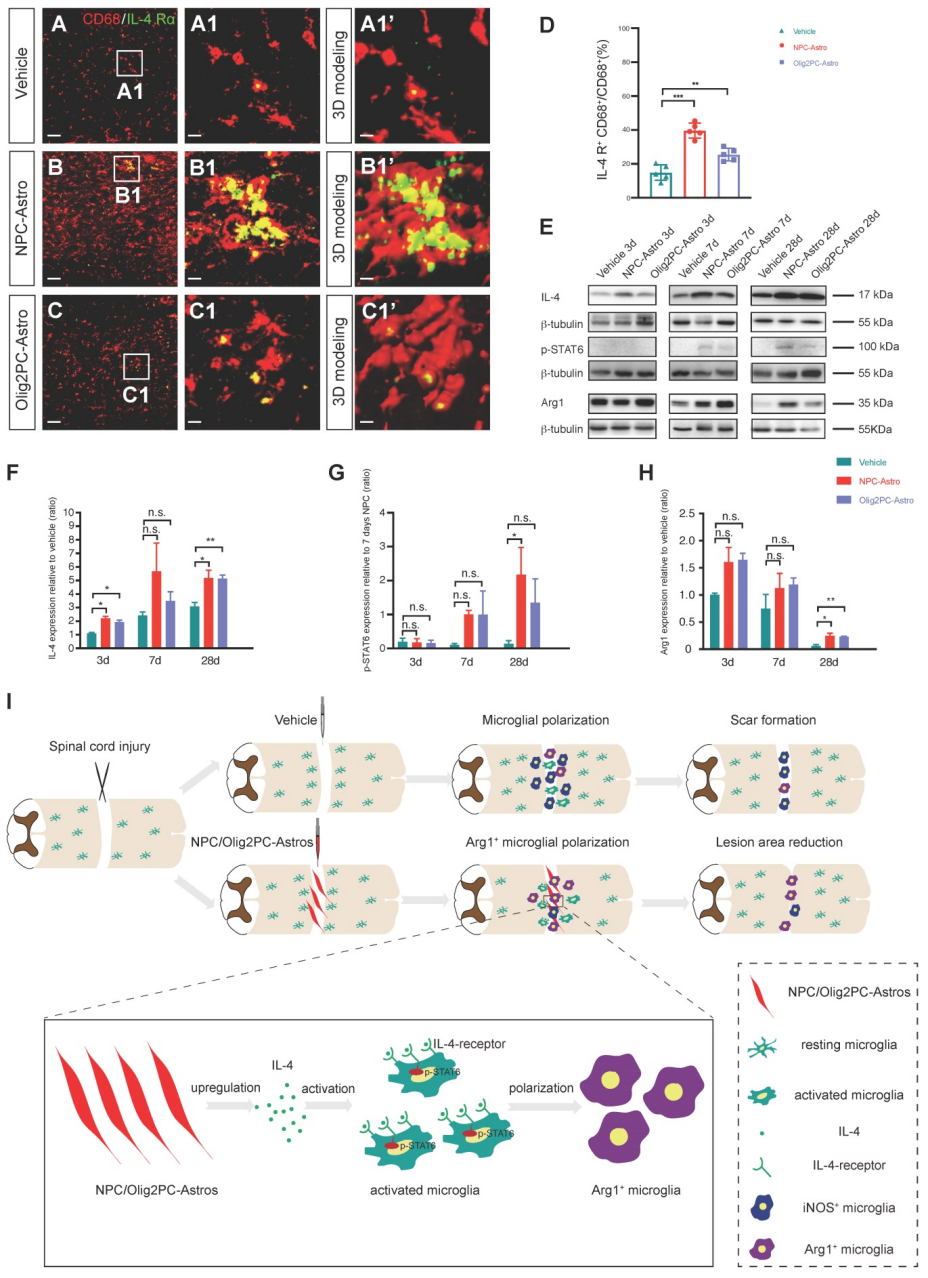
** Grafted astroglia activate anti-inflammatory microglia via IL-4 signaling. (A-C)** Representative images showing CD68 (red) and IL-4R (green) staining in the lesion center at 4 weeks post-transplantation. **A1-C1** are higher magnification images of boxed areas in **A-C**; **A1'-C1'** are three-dimensional models of the microglia in **A1-C1**. Scale bars: 30 μm **(A-C)**, 5 μm **(A1-C1, A1'-C1')**.** (D)** Quantification of IL-4R^+^CD68^+^ cells relative to all CD68^+^ cells (*n* = 5 per group). Brown-Forsythe and Welch ANOVA followed by Dunnett's T3 multiple comparisons test. Data are mean ± SD; **p < 0.01, ***p < 0.001. **(E)** Western blotting for IL-4, p-STAT6, and Arg1 expression in the lesion area at 3 to 28 days after transplantation.** (F-H)** Quantification of IL-4, p-STAT6 and Arg1 expression at 3 to 28 days (*n* = 3 per group). One-way ANOVA followed by Tukey's multiple comparisons test for each time point; once one-way ANOVA failed, Brown-Forsythe and Welch ANOVA followed by Dunnett's T3 multiple comparisons test or unpaired t test with Welch's correction. Data are mean ± SD; *p < 0.05, **p < 0.01, n.s., not significant. **(I)** Diagram depicting the model for anti-inflammatory polarization of microglia to promote repair of the spinal cord lesion after astroglial transplantation.

IL-4 has been identified as an agonist that promotes anti-inflammatory polarization of microglia 29, 30. The IL-4 receptor (IL-4R) is an IL-4-downstream molecule expressed on the plasma membrane of microglia, especially anti-inflammatory microglia 41. To examine the expression of IL-4R in activated microglia following astroglial transplantation, immunohistochemistry was carried out to detect IL-4R in CD68^+^ cells in the lesion area 4 weeks after transplantation. IL-4R expression in activated microglia was higher in the NPC-Astro and Olig2PC-Astro groups than in the vehicle group (**Figure 8A-C, A1-C1**). Three-dimensional modeling confirmed that IL-4R was expressed in microglia (**Figure 8A1'-C1'**). Quantification verified that the number of IL-4R^+^ microglia was higher in the NPC-Astro and Olig2PC-Astro groups than in the vehicle group (*n* = 5 per group, *p* < 0.001 and 0.01, respectively, **Figure 8D**). This indicated that the IL-4 pathway was activated by astroglial transplantation.

In inflammation-related diseases, IL-4 activates STAT6 via IL-4 receptors 1 and 2 (IL-4R1, IL-4R2) to regulate M1/M2 polarization via a STAT6-dependent pathway 42. The STAT6 pathway is a key signaling node for M2 (anti-inflammatory) activation of macrophages and microglia 43. Therefore, we employed western blotting to examine the expression of IL-4, p-STAT6, and Arg1 in the lesion area from 3 to 28 days after transplantation (**Figure 8E**). Compared with the vehicle group, IL-4 was significantly upregulated in the NPC-Astro group at 3 and 28 days (*n* = 3 per group, *p* < 0.05, **Figure 8F**), p-STAT6 was detectable at 7 days and significantly upregulated at 28 days (*n* = 3 per group, *p* < 0.05, **Figure 8G**), and Arg1 was upregulated at 28 days (*n* = 3 per group, *p* < 0.05, **Figure 8H**). Compared with the vehicle group, IL-4 was significantly upregulated in the Olig2PC-Astro group at 3 and 28 days (*n* = 3 per group, *p* < 0.05 and 0.01, **Figure 8F**), p-STAT6 was detectable at 7 days and 28 days, and Arg1 was significantly upregulated at 28 days (*n* = 3 per group, *p* < 0.01, **Figure 8H**). These results suggested that, following astroglial transplantation, IL-4 bound to IL-4R on activated microglia, thereby activating the STAT6 pathway and leading to a switch towards the anti-inflammatory microglial phenotype, as indicated by Arg1 expression.

As illustrated in **Figure 8I**, our results indicated that transplantation of NPC- and Olig2PC-Astros after SCI activated host microglia and increased their polarization to the anti-inflammatory (M2) phenotype. This change established an appropriate environment for lesion repair and axonal regeneration. In addition, our results revealed that anti-inflammatory microglia activation and polarization were mediated by activation of IL-4 signaling via upregulation of IL-4R expression on the microglial surface, which in turn upregulated p-STAT6.

## Discussion

Our results showed that grafted NPC- and Olig2PC-Astros survived in the lesion area and promoted the anti-inflammatory polarization of host microglia. The area of the lesion scar was reduced, and this was permissive for regrowth of serotonergic axons following experimental transection of the spinal cord. Furthermore, autonomous functional movements of the hindlimbs were partially restored. The reduction of lesion area and regrowth of serotonergic axons were both undetectable after depletion of anti-inflammatory microglia, confirming that host anti-inflammatory microglia played a key role in recovery. We further showed that polarization towards anti-inflammatory microglia was mediated by activation of IL-4 signaling. Our study is thus consistent with previous work showing the ability of specific astroglia to repair SCI and further provides a cellular and molecular mechanism.

Scars formed after SCI are composed of cell types with various origins (including reactive astrocytes 44, fibroblasts 17, a subset of pericytes 21, 45, microglia 24, and macrophages 26) and are believed to prevent axon regeneration as well as functional recovery 11, 46. Recent advances in understanding the composition and phenotypic characteristics of spinal injury scars have shown that targeting scar components enables improved functional outcomes after SCI 47. Reducing pericyte-derived scarring promotes regeneration of raphespinal and corticospinal axons and improves recovery of sensorimotor function 21. In neonatal mice, host microglia promote scar-free spinal cord healing and axon regeneration 28. By demarcating the border of the glial scar using GFAP and examining the expression of extracellular matrix components such as fibronectin and PDGFR-β, we found that transplantation with NPC- or Olig2PC-Astros reduced the area of the lesion scar and reduced deposition of fibronectin and PDGFR-β in the lesion area. We propose that this is permissive for regrowth of transected serotonergic axons and thus accounts for the observed improvement in motor function. Further, these extracellular matrix changes suggest that an increase in anti-inflammatory microglia may influence other components of scar formation at spinal cord lesions, offering opportunities for future investigation.

After injury, microglia proliferate extensively, accumulate at the lesion area, and polarize toward both pro-inflammatory and anti-inflammatory phenotypes (**Figure 8I**). Inhibiting pro-inflammatory polarization can alleviate necroptosis of oligodendrocytes and improve functional recovery in rats after SCI 48. Anti-inflammatory microglia suppress inflammation and exhibit neuroprotective effects through the secretion of anti-inflammatory cytokines 26, 29, 30. Our observations of a transition to highly activated anti-inflammatory microglia, of various cellular and functional improvements following astroglial transplantation, and loss of these improvements after depletion of microglia are all consistent with these established benefits of anti-inflammatory microglia.

Pathologically, the blood-spinal-cord barrier (BSCB) is disruptured after spinal cord injury 49, 50, and leads to the infiltration of peripheral inflammatory cells and factors into the lesion area 51. Recent studies have shown that peripheral macrophages affect the microenvironment of lesion area after SCI 52-54, and crosstalk between peripheral macrophages and vessels endothelial cells in the lesion area after SCI is a potential therapy for SCI 55. Moreover, ependymal cells can behave as multipotent spinal cord stem cells and form new neurons and glial cells 56-59, which may affect the microenvironment of lesion area after SCI. Our study focused on the tissue resident microglia response to astroglia transplantation after spinal cord injury. Due to the scope of our study and technical limitations, we can't exclude the possibility that cells from peripheral or stem cell after injury play roles on the repair of spinal cord injury. Actually, we have much interest in carrying out future studies relevant to this question.

Cytokines such as IL-4 29, 30 and IL-13 60 promote anti-inflammatory polarization of microglia, whereas IL-10 prevents proliferation of activated microglia/macrophages 61 as well as pathological hyperactivation of microglia 62. IL-4 promotes polarization of anti-inflammatory microglia by inducing phosphorylation of STAT6 and promoting transcription of STAT6-responsive genes such as Arg1 63. Consistent with these results, we observed an increase in Arg1^+^ anti-inflammatory microglia after astroglia transplantation as well as an increase in expression of IL-4, and correlated expression of p-STAT6. Hence, future analyses of IL-10 and IL-13 expression in the lesion area will be needed, and lineage tracing should be applied to better understand specific changes in microglial polarization and activation. Further knowledge of the balance between anti-inflammatory and pro-inflammatory microglial phenotypes in the lesion area may also help us better understand the complex immune microenvironment after SCI 29, 30.

## Conclusions

Our findings demonstrate that host anti-inflammatory microglia induced by transplantation with NPC- and Olig2PC-Astro have distinct roles in reducing the size of the lesion area and promoting axon regeneration and functional recovery. Our results define the mechanisms underlying activation and polarization of host microglia in the lesion area and emphasize the importance of astroglia and activated host anti-inflammatory microglia in promoting wound repair after SCI.

## Methods

### Animals

Adult female C57BL/6 mice (18-20 g, 8-10 weeks old) were used. All protocols were approved by the Animal Care and Use Committee at Soochow University.

Study design: This study was a parallel-designed randomized, double-blind trial. Intervention group was NPC-Astro or Olig2PC-Astro transplantation, vehicle group was PBS transplantation only, mice underwent laminectomy without spinal cord transection or astroglial transplantation as a sham control.

Sample size: The sample size was not estimated, because it was not chosen based on pre-specified effect size. We carried out multiple independent experiments using several biological replicates specified in figure legends. A total of 734 mice were used in this study. Among them, there were 236 mice in the NPC-Astro group or Olig2PC-Astro transplantation group, 236 mice in the control group and 26 mice in the sham group. Mice in sham group only used for BMS score (n=10), Western blotting (n=6), EMG detection (n=5) and gait analysis (n=5).

Mice were randomly assigned to experimental groups after SCI. All subjects all subjects survived until the end of the experiment, unless accidental death (Sham, n=0; Vehicle, n=13; NPC-Astro, n=10; Olig2PC-Astro, n=12).

### Spinal cord injury and cell grafting

Each mouse was anaesthetized with 2% (w/v) Avertin, and then laminectomy was performed at thoracic level 10 (T10). The dura was opened and the spinal cord completely transected using an iridectomy knife. Cell grafting was performed 1 day after surgery. We used TrypLE to disaggregate the cultured NPC-Astros or Olig2PC-Astros to single cell, and then quantified the astroglia number by cell counting chamber. After that, we centrifuged the astroglia and resuspended with PBS to a final concentration of 10^5^ cell per μl in PBS. The laminectomy site was exposed, and a pulled glass microcapillary pipette (Drummond^TM^, 5-000-1001-X10, 1 mm outside diameter, 0.5 mm inner diameter) was connected to a 10 μl Hamilton syringe and fixed on the stereotaxic frame (RWD, 78-8130). The pulled glass microcapillary pipette vertically inserted into the lesion site, and NPC-Astros or Olig2PC-Astros were transplanted at a concentration of 10^5^ cells/μl with four individual injections of 0.25 μl (± 0.2, ± 0.4 mm lateral to the midline, 0.5 mm below the dorsal surface of the spinal cord) at 0.25 μl/min. The pulled glass microcapillary was carefully removed to minimize leakage of the transplanted astroglia from the lesion site. As a vehicle control, mice were injected with the same volume of phosphate-buffered saline. For electromyography (EMG) and gait analysis, mice underwent laminectomy without spinal cord transection or astroglial transplantation as a sham control. All animals were placed on a heating pad after surgery. After laminectomy and throughout the study, all animals received daily subcutaneous injections of cyclosporine A (Millipore, 10 mg/kg), daily subcutaneous injections of ibuprofen (35 mg/kg) for pain relief and intramuscular injection of penicillin (10000 units per mouse) for the first week following surgery. Urine was expressed twice daily to prevent urinary tract infection.

### Astrocytes culture

Plates were coated with growth factor-reduced Matrigel (BD Biosciences). NPC- and Olig2PC-Astro cell lines, both derived from human embryonic stem cells, were established and provided by Dr. Wenbin Deng (Sun Yat-sen University) 5. NPC- and Olig2PC-Astros were cultured on separate plates in serum-free DMEM/F12 medium (Invitrogen) containing 1× N2 (Invitrogen), 1× B27 without retinoic acid (Invitrogen), 10 ng/ml BMP4 (Peprotech), and 20 ng/ml bFGF (Millipore). The medium was changed every 2 days until transplantation.

### Immunocytochemistry and immunohistochemistry

NPC- and Olig2PC-Astros were fixed with 4% (w/v) paraformaldehyde for 10 min, permeabilized with 0.2% (v/v) Triton X-100 for 10 min and blocked with 5% fetal bovine serum. Cells were incubated overnight at 4 °C with the following primary antibodies: monoclonal anti-S100β (Sigma-Aldrich cat. #S2532; RRID:AB_477499; 1:200), monoclonal anti-hN (Millipore cat. #MAB4383; RRID:AB_827439; 1:200), monoclonal anti-hM-Cy3 (Millipore cat. #MAB1273C3; RRID:AB_2631100; 1:500), or polyclonal anti-GFAP ( Millipore cat. #AB5804; RRID:AB_2109645; 1:200). After washing, they were incubated overnight at 4 °C with secondary antibodies conjugated with Alexa Fluor 488 or 555 (Invitrogen; 1:500).

Mice were anaesthetized with 4% (w/v) pentobarbital and perfused with 4% (w/v) paraformaldehyde. The spinal cord was post-fixed in 4% (w/v) paraformaldehyde overnight at 4 °C. Tissues were immersed serially in 20% and 30% (w/v) sucrose for cryosectioning. Immunohistochemistry was performed to assess astroglia survival, integration, neural protection, and axonal regeneration. Sections were incubated overnight at 4 °C with the following primary antibodies: monoclonal anti-hM-Cy3 (Millipore cat. #MAB1273C3; RRID: AB_2631100; 1:200), polyclonal anti-GFAP (Millipore cat. #AB5804; RRID: AB_2109645; 1:200), monoclonal anti-GFAP-Cy3 (Sigma-Aldrich cat. #C9205; RRID: AB_476889; 1:500), monoclonal anti-PDGFR-β (Abcam cat. #ab32570; RRID: AB_2262874; 1:200), monoclonal anti-fibronectin (Abcam cat. #ab2413; RRID: AB_777165; 1:200), polyclonal anti-serotonin (Sigma-Aldrich cat. #S5545; RRID: AB_477522; 1:500), monoclonal anti-synaptophysin (Millipore cat. #MAB5258; RRID: AB_2313839; 1:500), polyclonal anti-Iba1 (Abcam cat. #ab5076; RRID: AB_2224402; 1:500), polyclonal anti-iNOS (Abcam cat. #ab15323; RRID: AB_301857; 1:500), polyclonal anti-Arg1 (GeneTex cat. #GTX109242; RRID: AB_2036264; 1:500), polyclonal anti-MR (Abcam cat. #ab64693; RRID: AB_1523910; 1:500), polyclonal anti-Ki67 (Abcam cat. #ab15580; RRID: AB_443209; 1:500), or monoclonal anti-CD68 (Bio-Rad cat. #MCA1957; RRID: AB_322219; 1:500). After washing, sections were incubated overnight at 4 °C with secondary antibodies conjugated with Alexa Fluor 488, 555, or 647 (Invitrogen; 1:500) or Cy5 (Abcam cat. #ab6564; RRID: AB_955061; 1:500). DAPI (4',6-Diamidino-2-phenylindole; Sigma-Aldrich; 200 ng/ml) was used to stain cell nuclei.

### Assessment of wound healing

To view lesion sites, spinal cord tissue from mouse groups treated with vehicle, NPC-Astro, and Olig2PC-Astro was imaged with a digital surgical microscope (RWD, DOM-1001). For fluorescent labeling of the lesion site, spinal cord tissue was first made transparent using the CUBIC clearing technique 64-66, tissues were treated with reagent 1 for 4 days and reagent 2 for 3 days. The lesion area was then stained with anti-GFAP-Cy3 and imaged with a Zeiss LSM700 confocal microscope. In addition, parasagittal spinal cord sections were stained with anti-GFAP-Cy3 to outline the lesion area, anti-PDGFR-β to label pericytes, or anti-fibronectin to label fibroblasts. Five consecutive sagittal sections centered at the spinal cord midline were used to examine PDGFR-β^+^ or fibronectin^+^ area using ImageJ software (NIH Image).

### Quantification of axons

The regrowth of serotoninergic axons was measured using the serotonin (5-hydroxytryptamine, 5-HT) intensity of images taken with a Zeiss LSM700 confocal microscope under a 20× objective. A series of optical cross-sections were drawn at 0.1-mm intervals from the rostral lesion border (-1.0 to 0 mm) and caudal lesion border (0 to +1.0 mm) (**Figure 3 A**). The 5-HT intensity index at a specific position is a ratio of the intensity of sRST axons relative to the density of axons 1 mm rostral to the lesion border. The 5-HT intensity in each cross-section was calculated after subtracting background. At least three tissue slices per mouse were examined.

### Analysis of motor function

Recovery of hindlimb movement was evaluated weekly with an open-field test using the Basso Mouse Scale (BMS) according to the method developed by Basso and colleagues 67. BMS scores were evaluated by two independent investigators blinded to the identities of the groups.

The electromyogram (i.e., EMG) activity of the right hindlimb tibial anterior (TA) muscle was evaluated 4 weeks after astroglial transplantation. The recording electrode was fixed on the surface of the TA muscle, and the mouse was then fettered and kept in a vertical position on a treadmill 68. Voluntary hindlimb movements were captured when the treadmill speed reached 2.4 m/min. We collected the EMG data while the hindlimbs were positioned during standing for ~10 s in each group. EMG activity was recorded using a Biopac MP150 data acquisition system. All the amplitudes were measured and quantifited. Each data point in amplitude and interval was the mean of 10 cycles. The hip, knee, ankle, and foot joints of the right hindlimb were marked by small discs to reconstruct the trajectory of motion.

Gait analysis was performed 4 weeks after transplantation. Mice were trained to walk on a motor-driven treadmill at a constant speed of 5 cm/s for 20-s periods. An adjustable compartment measuring 17 cm × 5 cm was mounted over the treadmill belt to ensure that the mouse remained in the camera view at all times. TreadScan software (CleverSys) was used to correctly identify initial foot contact, base of support, stance duration, stride duration, foot liftoff, swing duration, stride length, track width, and toe spread data for each foot 69. Base of support and stride length were analyzed to evaluate hindlimb recovery.

### Analysis of microglial proliferation and phenotypic shift

Sholl analysis was performed to determine the activation state of microglia in the lesion area 70, 71. Microglia were identified using immunolabeling for Iba1 (total microglia), Arg1 (arginase-1; for anti-inflammatory microglia), and iNOS (inducible nitric oxide synthase; for pro-inflammatory microglia). Immunoreactivity was evaluated using images taken with a Zeiss LSM700 confocal microscope under a 20× objective on days 3, 7, 14, 28, and 56 after astroglial transplantation. “Total microglia” were defined as the number of microglia in a 2-mm-long region of spinal tissue around the lesion area. In addition, microglia in the lesion area were identified based on GFAP-defined lesion borders. Microglia in the lesion area were demarcated, and dashed lines were drawn at ±1.0 mm relative to the GFAP-defined lesion border.

We quantified the Iba1^+^ microglia with the “Analyze Particles” function of ImageJ software^72^. Firstly, we opened the images and clicked “Find Maxima”, and then adjusted the value of “Prominence” to determine the microglia position. Secondly, we inverted the images and converted them to 8-bit images, adjusted the thresholds to outline the cell contour, and then transformed the cell shape. Thirdly, we used image calculator to separate adhesive cells. Finally, we used “Analyze Particles” to count Iba1+ microglia. Two-way ANOVA followed by Tukey's multiple comparisons test was used for quantification of total number of microglia with GraphPad prism 8.2.0. All data were presented as a percentage of total microglia, iNOS^+^ microglia, or Arg1^+^ microglia in the vehicle group after subtracting background. At least three tissue slices per mouse were examined. The relative number of anti-inflammatory Arg1^+^ microglia was determined using Arg1 and Iba1 or CD68 immunoreactivity in images taken with a Zeiss LSM700 confocal microscope under a 20× objective 4 weeks after astroglial transplantation. At least three tissue slices per mouse were examined.

### Depletion of anti-inflammatory microglia

Mannosylated clodronate liposomes (MCLs), which deplete the pool of anti-inflammatory microglia 40, were injected into the lesion area along with astroglia or vehicle at 1 day post-SCI and then injected intraperitoneally once a week for 4 weeks after transplantation 40. The efficiency of anti-inflammatory microglial depletion was determined 4 weeks after transplantation by staining for Arg1^+^ microglia. PDGFR-β^+^ and fibronectin^+^ areas were evaluated 4 weeks after transplantation, and regrowth of descending serotonergic axons was evaluated 8 weeks after transplantation.

### Western blotting

Protein was isolated from a 2-mm-long region of spinal tissue containing the lesion area at 3, 7 and 28 days after transplantation. Western blotting was performed using standard SDS-PAGE, electroblotting, and enhanced chemiluminescence detection. Primary antibodies used for WB included: monoclonal anti-PDGFR-β (Abcam cat. #ab32570; RRID: AB_2262874; 1:5000), monoclonal anti-fibronectin (Abcam cat. #ab2413; RRID: AB_777165; 1:5000), monoclonal anti-IL-4 (Sino Biological cat. #11846-R404; 1:1000), monoclonal anti-phospho-STAT6 (CST cat. #56554; RRID: AB_2799514; 1:500), polyclonal anti-Arg1 (GeneTex cat. #GTX109242; RRID: AB_2036264; 1:1000), polyclonal anti-Iba1 (Abcam cat. #ab5076; RRID: AB_2224402; 1:1000). Secondary antibodies used were polyclonal Goat Anti-Mouse-HRP (Fdbio cat. #FDM007; 1:5000), polyclonal Rabbit anti-Goat-HRP (Fdbio cat. #FDG007; 1:5000) and polyclonal Goat Anti-Rabbit-HRP (Fdbio cat. #FDR007; 1:5000). Anti-β-tubulin (Fdbio cat. #FD0064; 1:5000) or anti-β-actin (Fdbio cat. #FD0060; 1:10000) was used as the loading control. Immunoreactivity was semi-quantitatively analyzed using ImageJ software. Images of blots were cropped for presentation.

### Statistical analysis

In Figure 2G, J, K, M, N, Figure 4J, K, N, O, Figure 5M, N, O, Figure 6D, J, L, Figure 8D, the quantification data conformed to the normal distribution but failed the test for homogeneity of variances, we performed the alternative Brown-Forsythe and Welch ANOVA followed by Dunnett's T3 multiple comparisons test. In Figure 6H, we performed unpaired t test with Welch's correction. In Figure 8F, G, H, we performed one-way ANOVA followed by Tukey's multiple comparisons test for each time point; once one-way ANOVA failed, the alternative Brown-Forsythe and Welch ANOVA followed by Dunnett's T3 multiple comparisons test or unpaired t test with Welch's correction was used. In Figure 3E. Figure 7G, H, Figure S1D, E, F, Figure S2D, E, G, Figure S3E-N and Figure S4D, E, I, J, the quantification data conformed to the normal distribution with homogeneous variances, we performed two-way ANOVA followed by Tukey's multiple comparisons test. In Figure 4A, B, Figure 5C, two-way ANOVA followed by Bonferroni's multiple comparisons test was performed. In Figure S1B, C, the quantification data conformed to normal distribution with homogeneous variances, and we performed one-way ANOVA followed by Tukey's multiple comparisons test. Data are presented as mean ± standard deviation (SD).

## Supplementary Material

Supplementary figures.

## Abbreviations

NPC-Astros: human ESC-derived neural progenitor cells
Olig2PC-Astros: Olig2-GFP knock-in progenitors
BMS: Basso Mouse Scale
SCI: spinal cord injury
FN: fibronectin
5-HT: 5-hydroxytryptamine
hN: human nuclei
hM: human mitochondria
EMG: electromyography
TA: tibial anterior
iNOS: inducible nitric oxide synthase
Arg1: Arginase 1
MR: mannose receptor
MCLs: mannosylated clodronate liposomes
IL-4: Interleukin 4
IL-4R: Interleukin 4 receptor
