# Supplementary material for: Grafted human ESC-derived astroglia repair spinal cord injury via activation of host anti-inflammatory microglia in the lesion area
Source: Theranostics. 2022 May 16;12(9):4288–309. doi: 10.7150/thno.70929 (PMC9169351; doi:10.7150/thno.70929)
Supplement: Supplementary file 1 — Supplementary figures. [file thnov12p4288s1.pdf]

Supplementary Figure 1

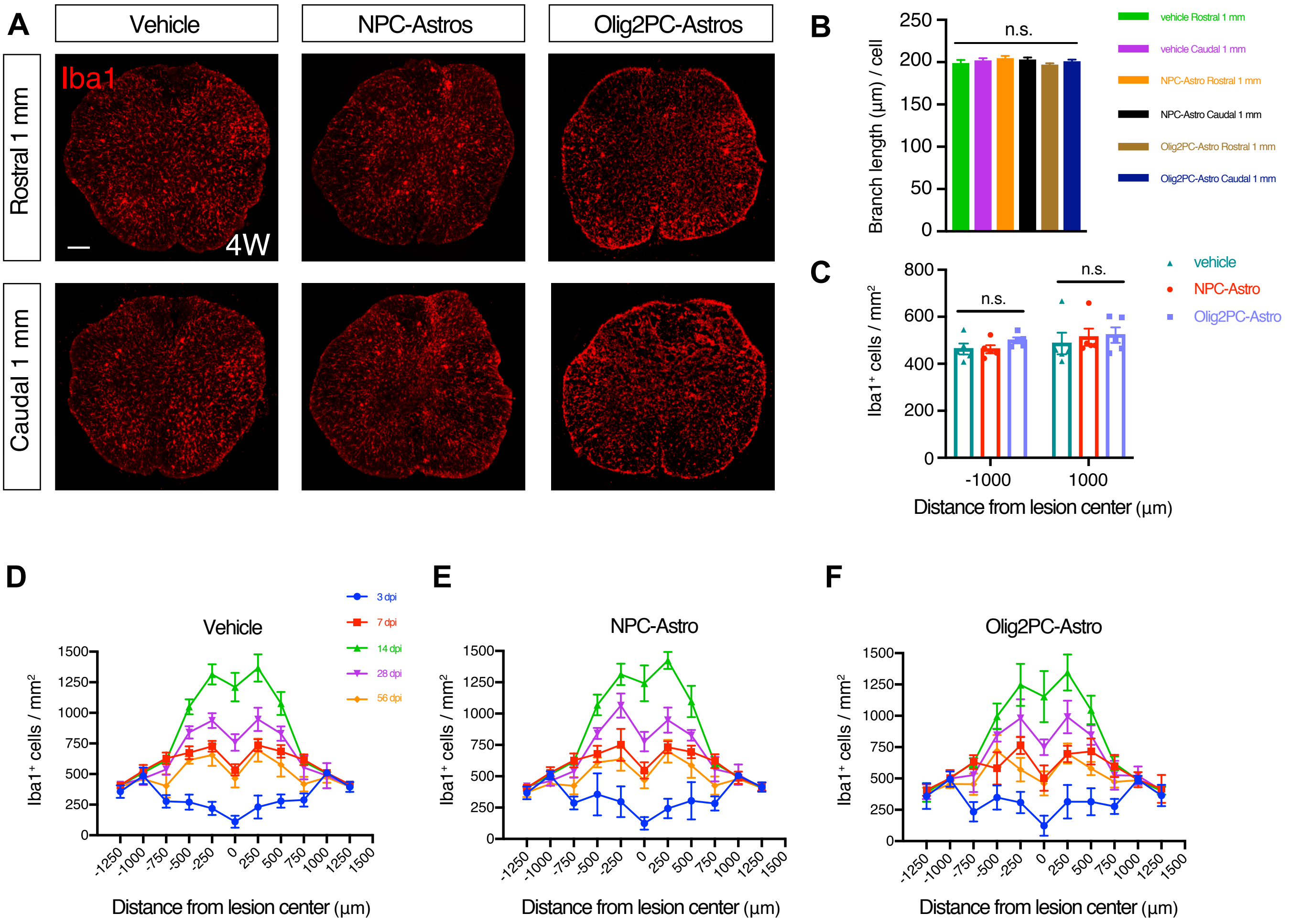

Figure S1: Microglia at rostral or caudal 1 mm showed no difference in the mumber and morphology.

(A) Confocal images of microglia in spinal cord sections taken both rostral (R) and caudal (C) 1 mm to the lesion epicenter. Scale bars: 100 μm. **(B)** Quantification of microglia branch in spinal cord sections taken both rostral (R) and caudal (C) 1 mm to the lesion epicenter 4 weeks after astroglia transplantation (*n* = 5 per group). One-way ANOVA followed by Tukey's test. **(C)** Quantification of total number of $Iba1^+$ microglia per mm^2 of tissue in spinal cord sections taken both rostral (R) and caudal (C) to the lesion epicenter at 4 weeks after astroglia transplantation (*n* = 5 per group). One-way ANOVA followed by Tukey's multiple comparisons test. **(D)** Quantification of the total number of $Iba1^+$ microglia per mm^2 of tissue in spinal cord sections taken both rostral (R) and caudal (C) to the lesion epicenter at 3-56 days after injury (*n* = 5 per group) **(E)** Quantification of the total number of $Iba1^+$ microglia per mm^2 of tissue in spinal cord sections taken both rostral (R) and caudal (C) to the lesion epicenter at 3-56 days after NPC-Astro transplantation (*n* = 5 per group). **(F)** Quantification of the total number of $Iba1^+$ microglia per mm^2 of tissue in spinal cord sections taken both rostral (R) and caudal (C) to the lesion epicenter at 3-56 days after Olig2PC-Astro transplantation (*n* = 5 per group). Two-way ANOVA followed by Tukey's multiple comparisons test. Data are mean ± SD; n.s., not significant.

Supplementary Figure 2

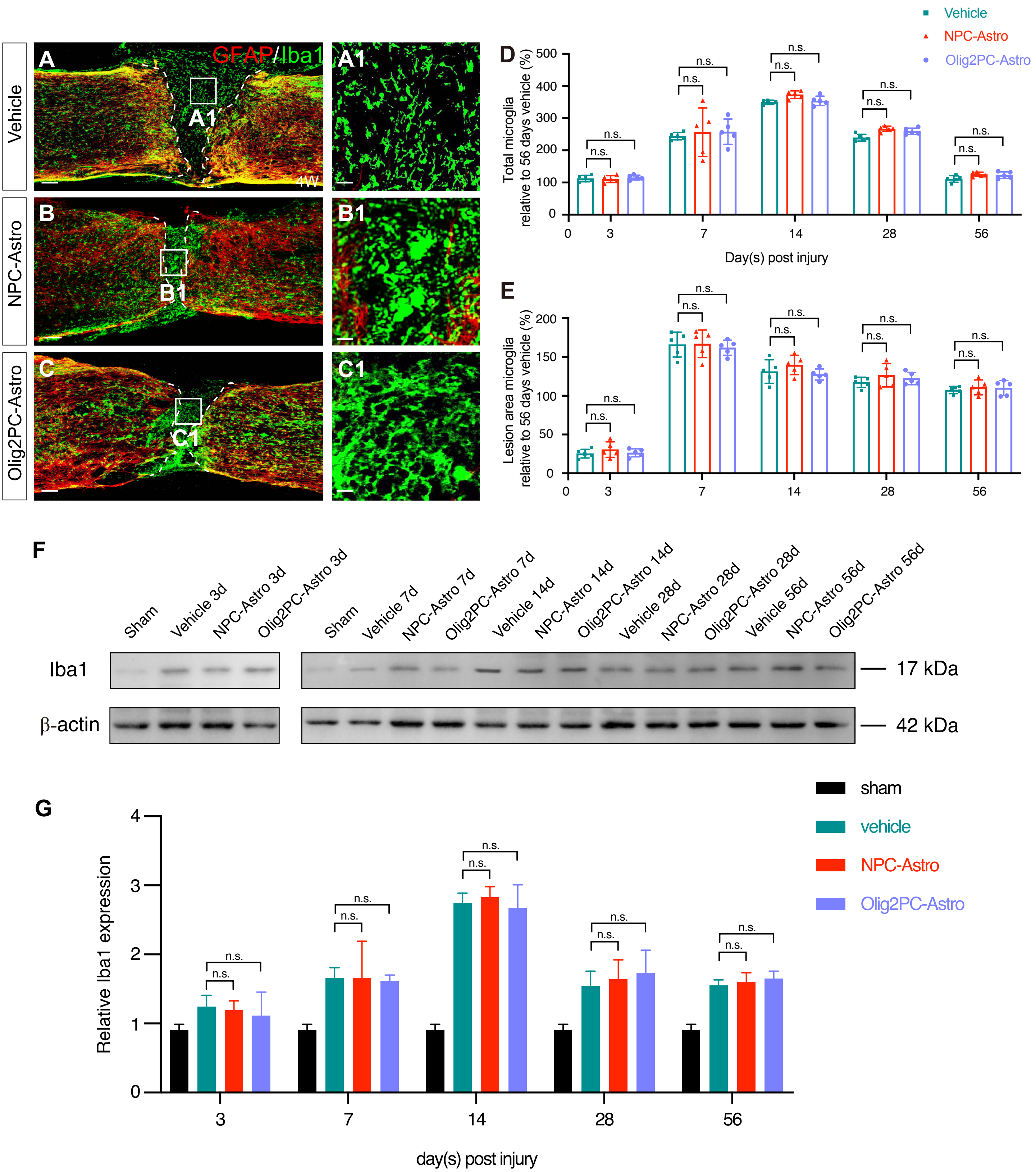

Figure S2: Grafted astroglia did not promote the increase of microglia.

(A-C) Representative images showing GFAP (red) and Iba1 (green) labeling in spinal cord sagittal sections at 4 weeks post-transplantation. **A1, B1, C1** are higher magnification images of boxed areas in **A-C**. Scale bars: 200 μm **(A-C)**, 20 μm **(A1-C1)**. **(D)** Quantification of total $Iba1^{+}$ microglia (*n* = 5 per group). **(E)** Quantification of $Iba1^{+}$ microglia in the lesion area (*n* = 5 per group). **(F)** Western blotting for Iba1 expression in the lesion area at 3 to 56 days after transplantation. **(G)** Quantification of Iba1 expression at 3 to 56 days (*n* = 3 per group). **D, E, G:** Two-way ANOVA followed by Tukey's multiple comparisons test. Data are mean ± SD; n.s., not significant.

Supplementary Figure 3

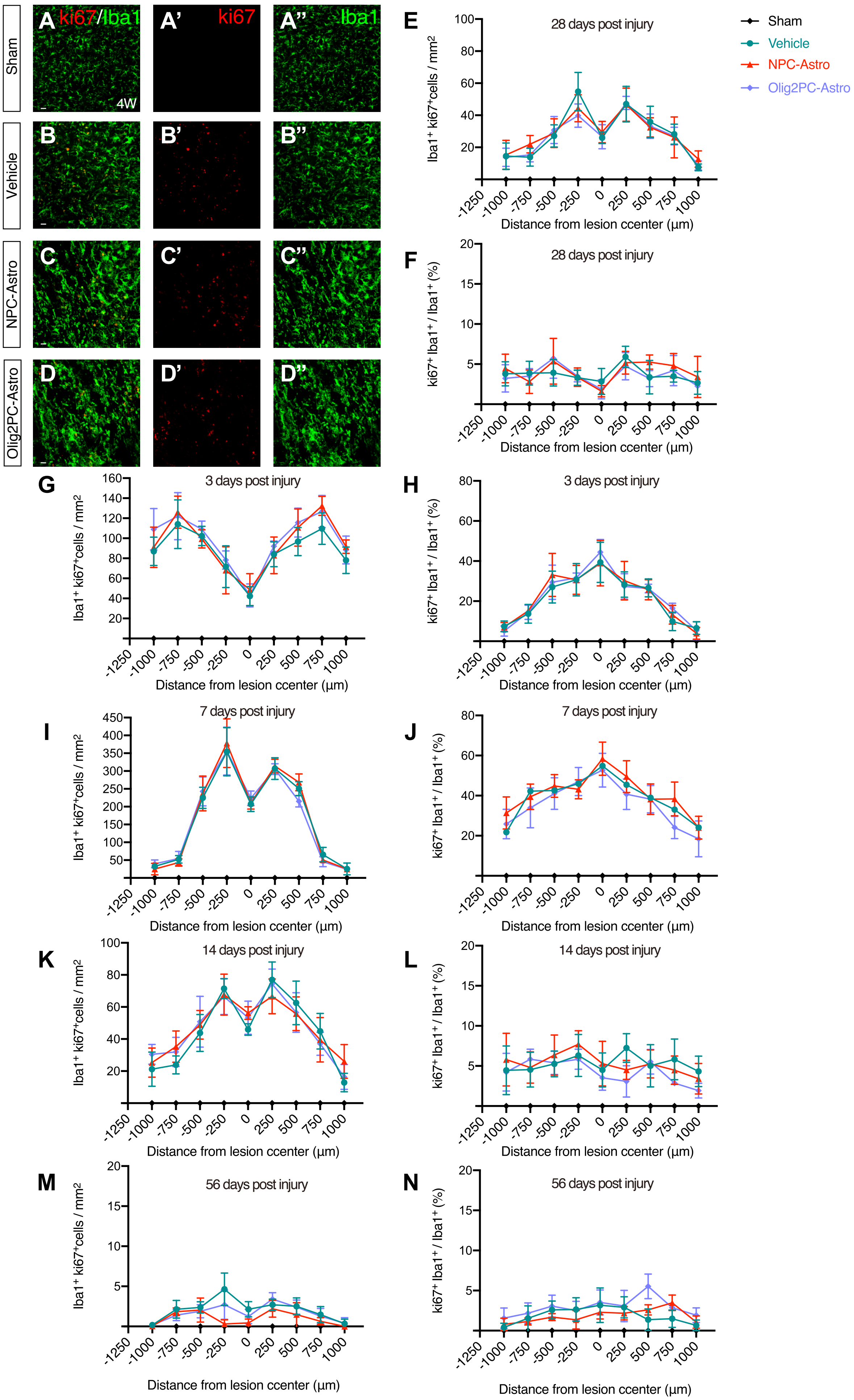

Figure S3: Grafted astroglia did not promote microglia proliferation.

(A-D) Representative images showing ki67 (red) and Iba1 (green) staining in the lesion center at 4 weeks post-transplantation. Scale bars: 20 μm. **(E)** Quantification of the number of actively proliferating microglia (Iba1$^+$ Ki67$^+$ cells) at 28 days after astroglia transplantation (n = 5 per group). **(F)** Percentage of Iba1$^+$ microglia undergoing proliferation 28 days after astroglia transplantation (n = 5 per group). **(G, I, K, M)** Quantification of the number of actively proliferating microglia (Iba1$^+$ Ki67$^+$ cells) at 3, 7, 14, 56 days after astroglia transplantation (n = 5 per group). **(H, J, L, N)** Percentage of Iba1$^+$ microglia undergoing proliferation 3, 7, 14, 56 days after astroglia transplantation (n = 5 per group). Two-way ANOVA followed by Tukey's multiple comparisons test. Data are mean ± SD; n.s., not significant.

Supplementary Figure 4

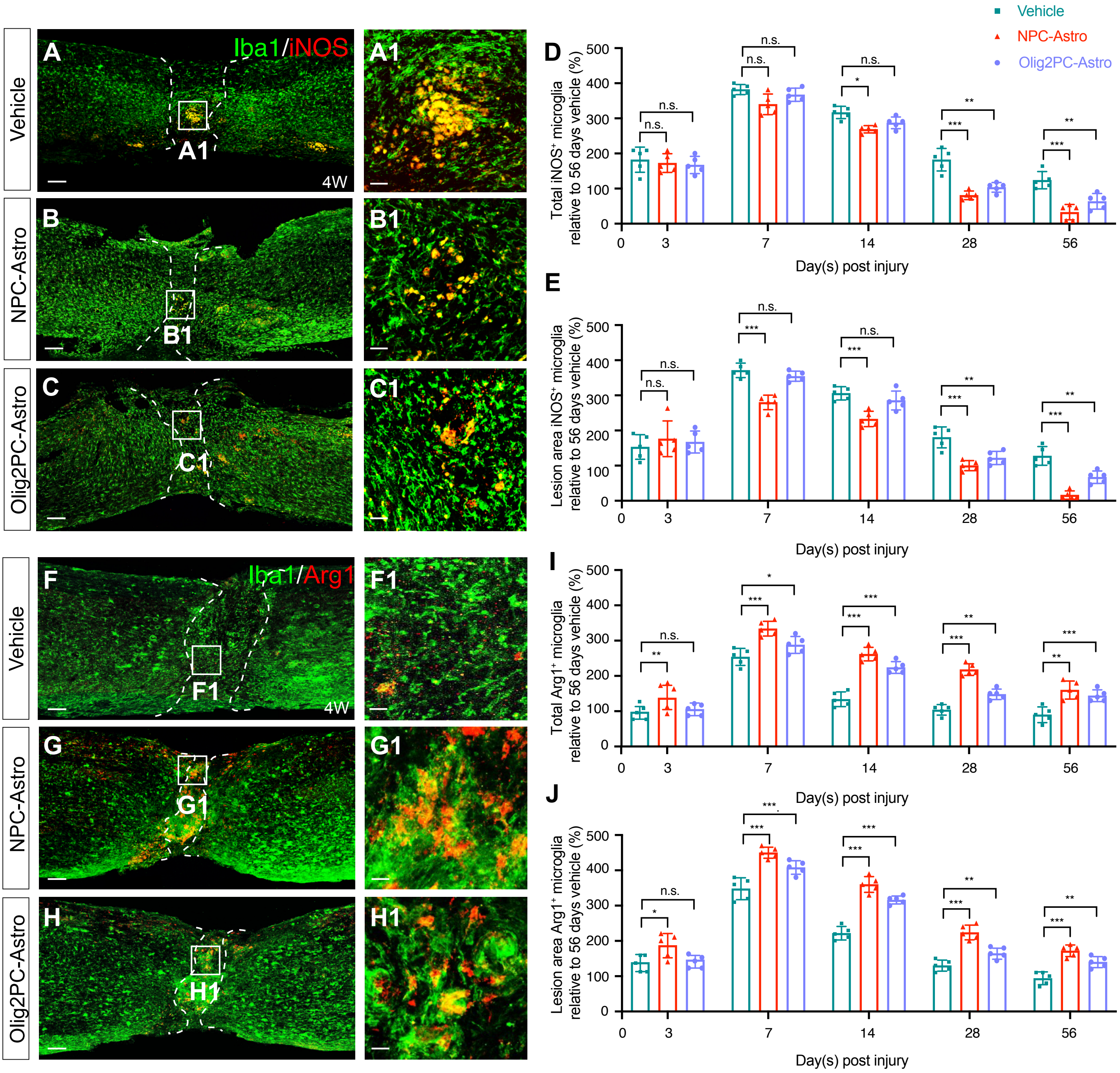

Figure S4: Grafted astroglia promote anti-inflammatory polarization of microglia. **(A-C)** Representative images showing Iba1 (green) and iNOS (red) labeling in spinal cord sagittal sections at 4 weeks post-transplantation. Scale bars: 200 μm **(A-C)**, 20 μm **(A1-C1)**. **(D)** Quantification of total $iNOS^+$ microglia (*n* = 5 per group). **(E)** Quantification of $iNOS^+$ microglia in the lesion area (*n* = 5 per group). **(F-H)** Representative images showing Iba1 (green) and Arg1 (red) labeling in spinal cord sagittal sections at 4 weeks post-transplantation. Scale bars: 200 μm **(F-H)**, 20 μm **(F1-H1)**. **(I)** Quantification of total $iNOS^+$ microglia (*n* = 5 per group). **(J)** Quantification of $iNOS^+$ microglia in the lesion area (*n* = 5 per group). **D, E, I, J:** Two-way ANOVA followed by Tukey's multiple comparisons test. Data are mean ± SD; $*p < 0.05$, $**p < 0.01$, $***p < 0.001$, n.s., not significant.
